# Metabolic-epigenetic axis in cardiovascular inflammatory injury: regulatory mechanisms and intervention strategies

**DOI:** 10.3389/fcvm.2026.1805737

**Published:** 2026-07-29

**Authors:** Xiaoyu Yu, Cong Cui, Jiyong He, Xiaoyu Guan, Zhe Zhang

**Affiliations:** 1 Liaoning University of Traditional Chinese Medicine, Shenyang, Liaoning, China; 2 The Affiliated Hospital of Liaoning University of Traditional Chinese Medicine, Shenyang, Liaoning, China

**Keywords:** cardiovascular inflammation, epigenetic regulation, ferroptosis, immunometabolism, metabolic reprogramming, traditional Chinese medicine

## Abstract

**Background:**

Cardiovascular diseases (CVDs) remain the leading cause of mortality worldwide, with inflammation central to disease progression. Emerging evidence reveals intricate crosstalk between metabolic reprogramming and epigenetic modifications in cardiovascular inflammatory injury, offering novel therapeutic strategies.

**Objective:**

This review elucidates the regulatory mechanisms underlying the metabolic–epigenetic axis in cardiovascular inflammation and explores both conventional and traditional Chinese medicine (TCM)–based intervention strategies.

**Results:**

Three findings are highlighted: (i) the succinate–hypoxia-inducible factor 1α (HIF-1α)–interleukin-1β (IL-1β) and itaconate–nuclear factor erythroid 2-related factor 2 (Nrf2) axes form an opposing pair defining macrophage polarization; (ii) acetyl-CoA, α-ketoglutarate (α-KG), lactate, and β-hydroxybutyrate (β-OHB) couple metabolism to histone modifications (H3K27ac, H3K27me3, H3K18la, H3K9ac) and DNA 5-hydroxymethylcytosine (5hmC), establishing trained immunity; (iii) mitochondrial dysfunction (mtDNA–cGAS-STING; mitochondrial ROS–NLRP3 inflammasome) and ferroptosis (GPX4/ACSL4) integrate these signals at the organelle level. TCM compounds (berberine, Shengmai Injection, tanshinone IIA, ginsenosides) modulate this axis at multiple nodes, with clinical support from the Phase III China Tongxinluo Study in Acute Myocardial Infarction (CTS-AMI) trial.

**Conclusions:**

The metabolic–epigenetic axis offers a tractable framework for precision anti-inflammatory cardiovascular therapy. Mechanistic studies and biomarker-guided trials are required to validate combined TCM-based interventions.

## 1 Introduction

Energy metabolism and epigenetic regulation interact closely in cardiovascular inflammation. This crosstalk modulates gene transcription and contributes to vascular and myocardial injury. Tricarboxylic acid (TCA) cycle intermediates serve dual functions, acting as critical mediators of immune responses beyond their metabolic roles (1). Among them, succinate and itaconate are two key regulators of macrophage inflammation that exert opposing effects.

Succinate is pro-inflammatory. During ischemia, succinate accumulates in tissues; upon reperfusion, it is rapidly oxidized and drives reactive oxygen species (ROS) generation through reverse electron transport at mitochondrial complex I, a primary mechanism of ischemia–reperfusion injury (2). Lipopolysaccharide (LPS) likewise induces succinate accumulation as macrophages shift from oxidative phosphorylation to glycolysis, and accumulated succinate stabilizes HIF-1α and promotes IL-1β transcription (3).

Itaconate, in contrast, is anti-inflammatory. Generated by cis-aconitate decarboxylase encoded by *IRG1*/*ACOD1*, itaconate inhibits succinate dehydrogenase, thereby coupling metabolic modulation to inflammation suppression (4), and also activates the Nrf2 antioxidant pathway by modifying cysteine residues on KEAP1 (5). Itaconate further restrains atherogenesis through Nrf2-dependent mechanisms; in human aortic specimens, ACOD1 expression correlates inversely with the severity of coronary stenosis (6).

Clinical studies have established inflammation as a key driver of cardiovascular outcomes. A collaborative analysis of statin-treated patients demonstrated that residual inflammatory risk, measured by high-sensitivity C-reactive protein (hsCRP), predicted future cardiovascular events more strongly than LDL cholesterol (7).

Anti-inflammatory therapies have demonstrated cardiovascular benefits in randomized trials. The CANTOS trial showed that canakinumab reduced major adverse cardiovascular events in post-myocardial infarction patients with elevated hsCRP, independent of changes in lipid levels (8), providing clinical validation for the succinate–HIF-1α–IL-1β arm of the metabolic–epigenetic axis. The COLCOT (9) and LoDoCo2 (10) trials reported similar benefits with colchicine, which engages a complementary node: by binding tubulin, colchicine prevents microtubule-dependent assembly of the NLRP3 inflammasome and suppresses mitochondrial ROS-driven IL-1β maturation, thereby targeting the mitochondrial-ROS arm rather than the transcriptional arm of the same axis.

Trained immunity refers to enhanced innate immune responses after initial activation and involves both metabolic reprogramming and epigenetic modifications (11). Hyperglycemia induces trained immunity in macrophages through glycolytic metabolism, with the transcription factor RUNX1 mediating accumulation of H3K4me3 and H3K27ac marks at the promoters of glycolytic and pro-inflammatory genes (12). Histone lactylation is another emerging mechanism that links metabolism to epigenetic regulation: during M1 macrophage polarization, H3K18la accumulates at later stages and drives *Arg1* expression, promoting a phenotypic shift toward an M2-like state (13).

Compounds derived from traditional Chinese medicine target immunometabolic pathways. Berberine (BBR) suppresses LPS-induced pro-inflammatory gene expression through AMPK activation (14) and attenuates atherosclerosis in *Apoe*-deficient mice via AMPK-dependent upregulation of UCP2 (15). At the clinical level, the Phase III CTS-AMI trial demonstrated that Tongxinluo capsules significantly reduced 30-day and 1-year major adverse cardiac and cerebrovascular events in patients with ST-elevation myocardial infarction (16).

This review elucidates the regulatory roles of metabolic intermediates in cardiovascular inflammation and the interplay between metabolic reprogramming and epigenetic modifications during trained immunity. Subsequent sections explore the regulation of epigenetic enzymes by metabolic substrates, the functional significance of histone lactylation in inflammatory cardiomyopathy, and therapeutic strategies—including TCM-based approaches—that target the metabolism–epigenetics axis.

## 2 Cell-Specific metabolic reprogramming in cardiovascular diseases

We argue that cell-type-specific metabolic reprogramming actively determines cardiovascular disease trajectory rather than serving as a passive byproduct. Three “metabolic switches”—glycolysis–TCA (tricarboxylic acid) disruption in macrophages, PFKFB3-driven aerobic glycolysis in endothelial cells, and substrate-flexibility loss in cardiomyocytes—share a common output: they regulate the supply of substrates available to chromatin-modifying enzymes. The evidence below tests whether each cellular phenotype is best explained by this metabolic–epigenetic coupling rather than by isolated bioenergetic deficits.

### 2.1 Overview of cell-specific metabolic reprogramming

Cardiovascular diseases (CVDs) arise from intercellular crosstalk in which metabolic adaptation is strongly cell-type-specific (17). Macrophages, endothelial cells, and cardiomyocytes each undergo distinct metabolic transitions that actively drive pathological progression. High-throughput approaches—single-cell sequencing, transcriptomics, and metabolomics—have established that metabolites act as direct regulators of gene expression, chromatin state, and immune activation rather than as passive energetic substrates (18).

**Figure 1:**
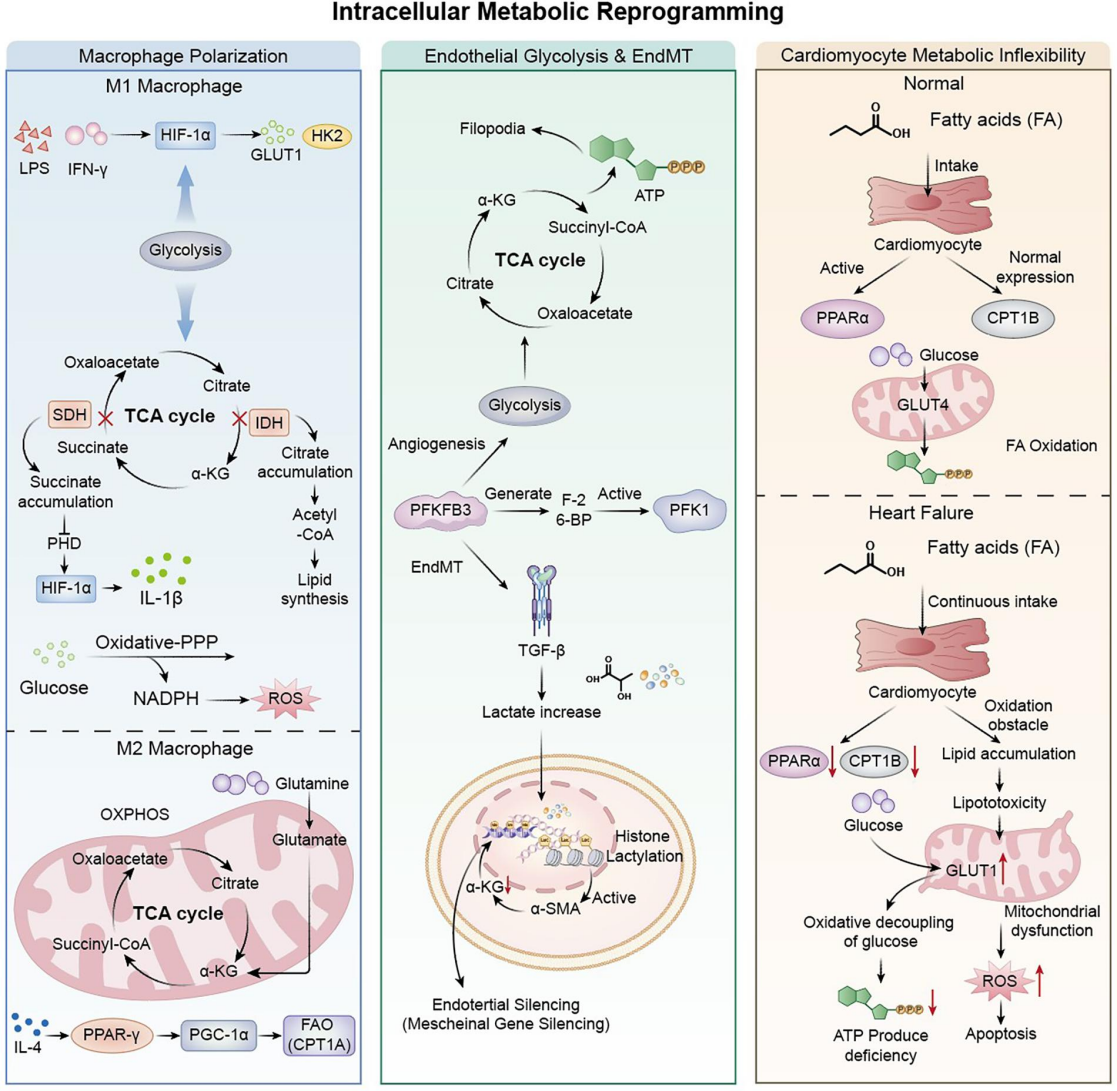
Cell-type-specific intracellular metabolic reprogramming in cardiovascular cells. Macrophages, endothelial cells, and cardiomyocytes each operate a distinct “metabolic switch” that determines their inflammatory and functional output in cardiovascular disease. **(A)** Macrophage polarization. Upon LPS/IFN-γ stimulation, M1 macrophages activate HIF-1α-driven aerobic glycolysis with concomitant upregulation of GLUT1 and hexokinase 2 (HK2). The TCA cycle is interrupted at two nodes—isocitrate dehydrogenase (IDH) and succinate dehydrogenase (SDH)—producing citrate accumulation that fuels acetyl-CoA generation and lipid synthesis, and succinate accumulation that competitively inhibits prolyl hydroxylase (PHD), stabilizing HIF-1α and driving *Il1b* transcription. The oxidative pentose phosphate pathway concurrently generates NADPH for sustained ROS production. In contrast, IL-4-induced M2 macrophages rely on oxidative phosphorylation (OXPHOS) and fatty acid oxidation (FAO), with PPARγ–PGC-1α-mediated upregulation of CPT1A; glutaminolysis-derived α-ketoglutarate (α-KG) replenishes the TCA cycle and supports tissue-repair gene expression. **(B)** Endothelial glycolysis and endothelial-to-mesenchymal transition (EndMT). Endothelial cells (ECs) rely predominantly on PFKFB3-driven aerobic glycolysis to sustain angiogenesis: PFKFB3 generates fructose-2,6-bisphosphate (F-2,6-BP), allosterically activating phosphofructokinase-1 (PFK1) to fuel ATP production at filopodia and lamellipodia. Under TGF-β signaling, glycolytic output rises and lactate accumulation drives p300-catalyzed histone lysine lactylation (H3K18la), activating mesenchymal gene expression (α-smooth muscle actin, α-SMA), while concurrent α-KG depletion silences endothelial-identity genes through impaired TET-mediated DNA demethylation, jointly executing the EndMT program. **(C)** Cardiomyocyte metabolic inflexibility. Healthy adult cardiomyocytes derive ∼90% of ATP from FAO through PPARα-licensed CPT1B activity, with glucose uptake via GLUT4 contributing the remainder. In heart failure, sustained pathological stress suppresses PPARα and CPT1B expression, impairing mitochondrial FAO and producing intracellular lipid accumulation and lipotoxicity. Compensatory upregulation of GLUT1 increases glucose uptake but oxidative coupling fails, producing ATP-generation deficiency, mitochondrial dysfunction with elevated ROS, and ultimately cardiomyocyte apoptosis. α-KG, α-ketoglutarate; α-SMA, α-smooth muscle actin; CPT1A/B, carnitine palmitoyltransferase 1A/B; ECs, endothelial cells; EndMT, endothelial-to-mesenchymal transition; F-2,6-BP, fructose-2,6-bisphosphate; FA, fatty acids; FAO, fatty acid oxidation; GLUT1/4, glucose transporter 1/4; H3K18la, histone H3 lysine 18 lactylation; HIF-1α, hypoxia-inducible factor-1α; HK2, hexokinase 2; IDH, isocitrate dehydrogenase; IFN-γ, interferon-γ; IL-1β/4, interleukin-1β/4; LPS, lipopolysaccharide; OXPHOS, oxidative phosphorylation; PFK1, phosphofructokinase-1; PFKFB3, 6-phosphofructo-2-kinase/fructose-2,6-bisphosphatase 3; PGC-1α, peroxisome proliferator-activated receptor-γ coactivator-1α; PHD, prolyl hydroxylase; PPARα/γ, peroxisome proliferator-activated receptor-α/γ; PPP, pentose phosphate pathway; ROS, reactive oxygen species; SDH, succinate dehydrogenase; TCA, tricarboxylic acid; TGF-β, transforming growth factor-β.

Metabolic dysfunction may also represent disruption of metabolite-mediated intercellular signaling—macrophage-derived metabolites influence neighboring endothelial cells, and altered endothelial metabolism modifies cardiomyocyte substrate use through local signaling (19). Figure 1 summarizes the characteristic pattern of each cell type: HIF-1α/SDH-driven glycolytic remodeling vs. OXPHOS (oxidative phosphorylation)/FAO (fatty acid oxidation) restoration in M1 vs. M2 macrophages; PFKFB3-driven aerobic glycolysis and TGF-β/lactate-driven EndMT (endothelial-to-mesenchymal transition) in endothelial cells; and PPARα/CPT1B suppression with compensatory GLUT1-mediated glucose uptake in failing cardiomyocytes.

### 2.2 Cell type-specific metabolic reprogramming

#### 2.2.1 Metabolic polarization of macrophages: the switch between Pro-inflammatory and reparative states

M1 (pro-inflammatory) and M2 (anti-inflammatory) polarization of cardiovascular macrophages involves extensive metabolic reprogramming that directly links macrophage function to substrate use (20).

##### 2.2.1.1 Glycolytic reprogramming and TCA disruption in M1 macrophages

LPS (lipopolysaccharide) or IFN-γ stimulation upregulates aerobic glycolysis (a Warburg-like effect) in M1 macrophages despite adequate oxygen, supplying biosynthetic intermediates and signaling molecules for inflammatory output (21). The pentose phosphate pathway is activated in parallel, providing NADPH for both anabolism and NADPH oxidase-driven ROS (reactive oxygen species) that regulate inflammatory gene expression (22).

M1 polarization also produces selective TCA-cycle interruption: citrate accumulates as IDH (isocitrate dehydrogenase) activity falls, and succinate accumulates as SDH (succinate dehydrogenase) activity falls. Mills et al. demonstrated that LPS-stimulated macrophages shift from OXPHOS to glycolysis, with SDH-driven succinate oxidation and elevated mitochondrial membrane potential together generating ROS that activates pro-inflammatory transcription; pharmacological SDH inhibition (dimethyl malonate) lowers IL-1β and raises IL-10 (23). Glutamine-derived anaplerotic succinate then stabilizes HIF-1α, a direct transcriptional driver of IL-1β (3); HIF-1α in turn upregulates glycolytic genes (GLUT1, hexokinases, LDH), creating a self-amplifying loop between glycolysis and HIF-1α (24). The CANTOS trial—in which IL-1β neutralization reduced major adverse cardiovascular events in patients with residual inflammatory risk—provides clinical confirmation of this immunometabolic circuit (8).

Citrate accumulation has a separate epigenetic consequence: cytosolic citrate is cleaved by ACLY (ATP-citrate lyase) to acetyl-CoA, which serves both as a lipid precursor and as substrate for histone acetylation, thereby linking glucose flux to inflammatory gene transcription (25).

##### 2.2.1.2 Oxidative metabolism in M2 macrophages

IL-4 or IL-13 activation drives M2 polarization through CPT1A-mediated FAO coupled to OXPHOS (26). PPARγ is the central transcriptional regulator: myeloid-specific *Pparγ* deletion impairs alternative activation and worsens diet-induced obesity and insulin resistance in mice, indicating a metabolically protective role for tissue-resident M2-like macrophages (27). After myocardial infarction, M2 macrophages support angiogenesis, extracellular matrix remodeling, and reparative fibrosis (28). Glutamine-derived α-ketoglutarate (α-KG) is required for M2 polarization through FAO and Jmjd3-dependent histone demethylation; the α-KG/succinate ratio is directional, with high values favoring M2 and low values favoring M1 (29).

The M1/M2 distinction itself represents a continuum rather than a binary state. Single-cell RNA sequencing of aortic macrophages has identified resident-like populations in both healthy and atherosclerotic aortae, together with inflammatory (IL-1β-high) and lipid-handling TREM2⁺ subsets that expand in disease (30).

#### 2.2.2 Endothelial glycolysis dependence and endothelial-to-mesenchymal transition

Endothelial cells maintain vascular homeostasis and mediate immune trafficking, deriving most of their ATP from glycolysis even when oxygen is plentiful—the endothelial Warburg effect (31).

##### 2.2.2.1 Physiological basis and PFKFB3 in pathological angiogenesis

Mitochondria occupy only a small fraction of endothelial cell volume, so a glycolysis-dominant strategy preserves O₂ for NO synthesis and other oxygen-dependent signaling (31). Approximately 85% of endothelial ATP is glycolytically derived, and glycolytic enzymes associate with cytoskeletal elements to form compartmentalized “metabolic hotspots” (32). PFKFB3 sustains vessel sprouting: De Bock et al. reported that endothelial glycolytic flux exceeds OXPHOS, β-oxidation, and glutaminolysis by more than two orders of magnitude, and that PFKFB3 loss disrupts filopodia dynamics, tip-cell selection, and Notch-regulated stalk-cell behavior (33).

Under inflammation, hyperglycemia, or hypoxia, PFKFB3 is markedly upregulated. Excess flux generates toxic methylglyoxal, drives protein glycation, and competitively restricts the pentose phosphate pathway, depleting NADPH (34). The PFKFB3 inhibitor 3PO reduces pathological neovascularization across multiple disease models—oxygen-induced retinopathy, psoriasis, and colitis—without abolishing physiological angiogenesis, identifying PFKFB3 as a tractable target in neovascular disease (35).

##### 2.2.2.2 Metabolic drivers of endothelial-to-mesenchymal transition (EndMT)

During EndMT, endothelial cells acquire mesenchymal features and contribute to atherosclerotic plaque biology, pulmonary vascular remodeling, and cardiac fibrosis (42). TGF-β–induced EndMT downregulates CPT1A and suppresses FAO; FAO restrains EndMT by modulating intracellular acetyl-CoA and SMAD7 signaling, and endothelial-specific *Cpt2* deletion in mice worsens EndMT and increases vascular permeability (43). FAO is therefore required for endothelial phenotypic stability.

#### 2.2.3 Cardiomyocyte metabolic inflexibility in heart failure

The heart accounts for only ∼0.5% of body weight but consumes ∼10% of resting energy expenditure (36). The healthy adult myocardium derives ∼90% of ATP from FAO with smaller contributions from glucose, lactate, and ketones, and switches between substrates according to availability and demand (37). In heart failure (HF), this flexibility is lost and the heart shifts toward less efficient pathways (17).

##### 2.2.3.1 Substrate inflexibility and fetal gene reactivation

Under chronic pathological stress, cardiomyocytes recapitulate a fetal-like program in which FAO genes (CPT1B, LCAD) are repressed and glucose-utilization genes (GLUT1, hexokinase) are upregulated (36). PPARα, the master transcription factor for FAO genes, is downregulated, and the Randle cycle—in which FAO-derived acetyl-CoA and citrate inhibit pyruvate dehydrogenase, and vice versa—is pathologically biased (38). Studies of explanted failing human hearts indicate that disordered intermediary metabolism and oxidative flux, rather than absolute ATP depletion, are the primary drivers of contractile failure (36).

##### 2.2.3.2 Lipotoxic intermediates and bioenergetic collapse

Imbalance between fatty acid uptake and oxidation produces a lipotoxic signature dominated by directional shifts in specific lipid species rather than indiscriminate “lipid overload” in the failing myocardium. Long-chain ceramides (C16:0, C18:0, C20:0) and serine palmitoyltransferase (SPT) are elevated in tissue and circulation, and SPT inhibition with myriocin or *SPTLC2* deletion in mice attenuates post-infarction remodeling (39). Long-chain ceramides correlate with myocardial infarction, HF, and cardiovascular mortality in human cohorts, whereas very-long-chain species (C22:0, C24:0) appear neutral or protective (40). Mechanistically, ceramides activate PP2A and atypical PKC*ζ*, dephosphorylate Akt, and—in the case of adipocyte-derived C16:0—drive eNOS uncoupling and vascular oxidative stress (41).

Diacylglycerol (DAG) also accumulates, and PKCα and PKC*ε* phosphorylate cardiac troponin I and cMyBP-C, reducing myofilament Ca^2+^ sensitivity (42). Long-chain acylcarnitines accumulate when β-oxidation cannot match fatty acid input, independently predict mortality in chronic HF patients, and decline after mechanical unloading (43, 44). The inner-mitochondrial-membrane phospholipid tetralinoleoyl cardiolipin (L_4_CL) is progressively lost in failing hearts, destabilizing respiratory supercomplexes (45). The combined effect of elevated long-chain ceramides, increased DAG, accumulating acylcarnitines, and depleted L₄CL is impaired oxidative phosphorylation, with ketone-body (β-hydroxybutyrate) oxidation rising as a compensatory fuel (46).

Disordered mitochondrial dynamics—excessive DRP1-driven fission with reduced MFN1/2- and OPA1-mediated fusion—compounds these lipotoxic and bioenergetic defects and is linked to impaired mitophagy (47–49). Section 3 develops the broader mitochondrial machinery that connects these phenotypes to innate immunity.

##### 2.2.3.3 Therapeutic strategies targeting cardiac metabolism

The partial FAO inhibitor trimetazidine shifts substrate use toward glucose by blocking long-chain 3-ketoacyl-CoA thiolase (50). and ranolazine produces a comparable substrate shift in normoxic, ischemic, and reperfused hearts (51). Ketone bodies serve both as alternative fuel and as signaling molecules: in chronic HF patients, infused 3-hydroxybutyrate acutely raises cardiac output by ∼40% and stroke volume by ∼30% without lowering mechanical efficiency, providing first-in-human evidence for direct hemodynamic benefit (52). SGLT2 inhibitors raise circulating ketone availability and reduced cardiovascular death or HF hospitalization across diabetic and non-diabetic HFrEF populations in DAPA-HF (53). Vericiguat restores depressed NO–cGMP–PKG signaling and reduced cardiovascular death or first HF hospitalization in HFrEF (54); omecamtiv mecarbil increases contractility without raising cytosolic Ca^2+^ and produced a smaller but significant benefit on the same composite endpoint (55). Metformin, through AMPK activation, modifies cardiomyocyte substrate use and was associated with improved clinical outcomes in an Alberta cohort of patients with diabetes and HF (56), and similarly with lower mortality in a separate ambulatory cohort (57). Mitochondria-anchored agents complement these substrate-shifting strategies: the cardiolipin-stabilizing peptide elamipretide improves complex I/IV activity and supercomplex assembly in failing human hearts (58), and MitoQ accumulates in the matrix and reduces lipid peroxidation downstream of complex I/III superoxide leak (59).

An open question remains: if every cardiovascular cell type carries its own metabolic signature, can pharmacologically reprogramming one cell type—for example, restoring cardiomyocyte FAO—propagate beneficial epigenetic changes to neighboring macrophages and endothelial cells, or are cell-autonomous interventions required?

## 3 Mitochondria as an integrator of metabolic stress and innate immunity

In cardiovascular disease, mitochondria are not bystanders—they translate metabolic stress into innate immune activation. We argue that three apparently distinct mitochondrial events—dynamics imbalance, mtDNA release engaging cGAS–STING, and mtROS-dependent NLRP3 inflammasome assembly—share a common mechanistic spine: TCA-cycle metabolites. Succinate, itaconate, fumarate, and α-ketoglutarate (α-KG) act simultaneously as inflammatory signals and as cofactors for epigenetic enzymes. Read this way, “mitochondrial dysfunction” represents one metabolic–epigenetic node rather than a set of parallel injury pathways. The subsections below test that proposition.

**Figure 2:**
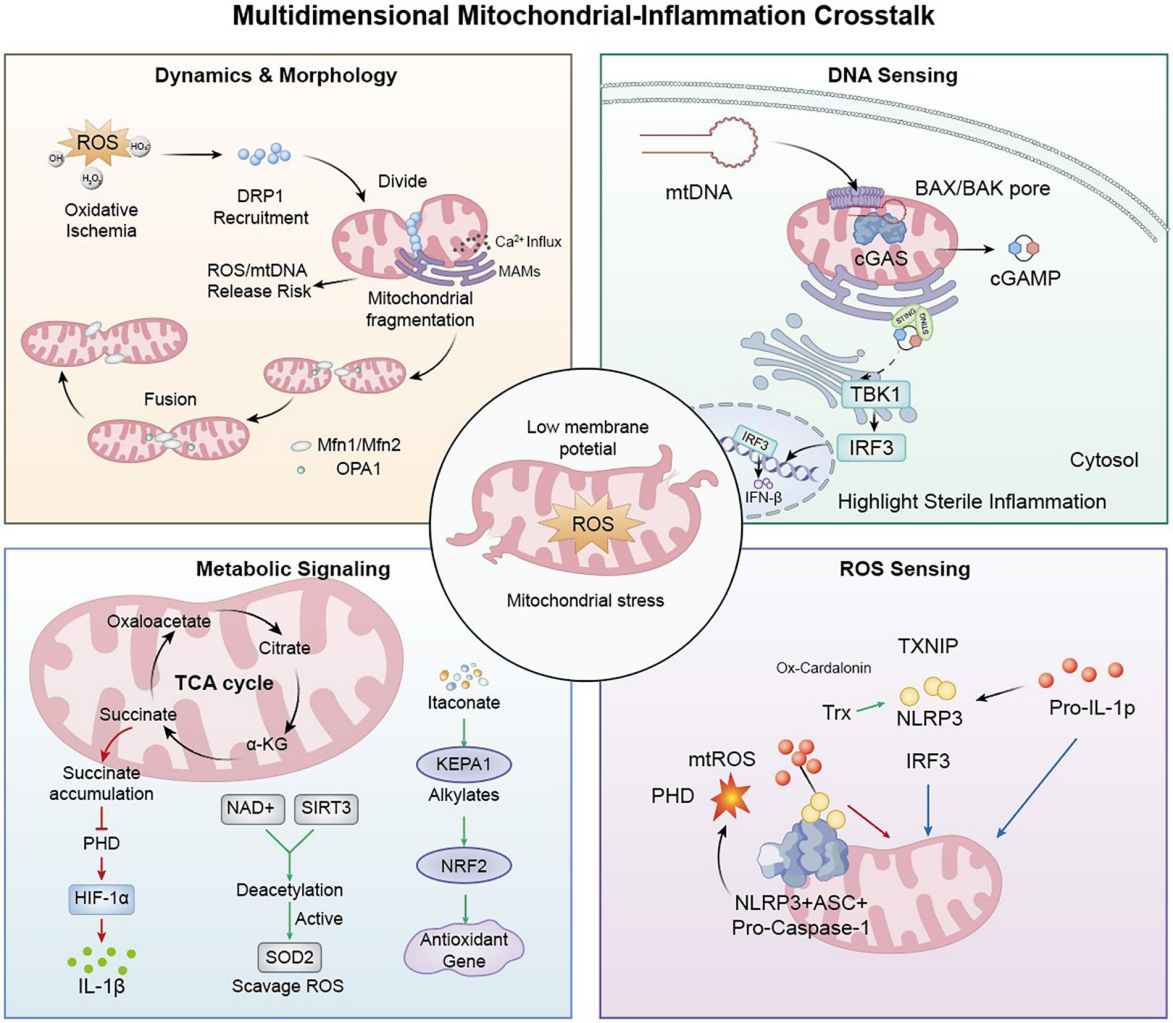
Multidimensional mitochondrial–inflammation crosstalk: mitochondria as the central hub integrating metabolic stress and innate immune activation. Under cardiovascular metabolic stress—characterized by mitochondrial membrane potential collapse and ROS overproduction—mitochondria initiate sterile inflammation through four mechanistically distinct but functionally convergent pathways. **(A)** Dynamics and morphology. Oxidative and ischemic stress recruits dynamin-related protein 1 (DRP1) to mitochondria-associated ER membranes (MAMs), where Ca^2+^ influx and DRP1 oligomerization drive excessive fission. Resulting mitochondrial fragmentation increases the risk of mtROS and mtDNA release, while compensatory MFN1/MFN2/OPA1-mediated fusion provides protective remodeling. **(B)** DNA sensing. Mitochondrial outer membrane permeabilization through BAX/BAK macropores—or mitochondrial permeability transition pore (mPTP) opening—releases mtDNA into the cytosol. Cytosolic mtDNA is sensed by cyclic GMP–AMP synthase (cGAS), generating 2′,3′-cGAMP, which activates STING at the ER–Golgi interface. STING recruits TANK-binding kinase 1 (TBK1), driving IRF3 phosphorylation, nuclear translocation, and type I interferon (IFN-β) transcription, thereby establishing sterile inflammation. **(C)** ROS sensing. Mitochondrial ROS (mtROS) triggers NLRP3 inflammasome assembly through two parallel mechanisms: TXNIP dissociation from oxidized thioredoxin (Trx) and cardiolipin externalization to the mitochondrial outer membrane, both providing molecular scaffolds for NLRP3 oligomerization with ASC and pro-caspase-1. The activated inflammasome cleaves pro-IL-1β into mature IL-1β, while IRF3 signaling provides parallel transcriptional input. **(D)** Metabolic signaling. TCA-cycle remodeling generates immunometabolic mediators with opposing functions. Succinate accumulation competitively inhibits PHD, stabilizing HIF-1α and driving *Il1b* transcription, reinforcing the pro-inflammatory output. Conversely, itaconate—generated by IRG1/ACOD1—alkylates KEAP1 cysteine residues to release Nrf2, driving antioxidant gene expression. NAD⁺-dependent SIRT3 deacetylates superoxide dismutase 2 (SOD2), enhancing mitochondrial ROS scavenging. These metabolite-driven anti-inflammatory feedback loops counterbalance the pro-inflammatory arms and define mitochondria as a single integrative metabolic–epigenetic node rather than parallel injury pathways. ASC, apoptosis-associated speck-like protein containing a CARD; BAX/BAK, Bcl-2-associated X protein/Bcl-2 antagonist or killer; cGAMP, 2′,3′-cyclic GMP–AMP; cGAS, cyclic GMP–AMP synthase; DAMPs, damage-associated molecular patterns; DRP1, dynamin-related protein 1; HIF-1α, hypoxia-inducible factor-1α; IFN-β, interferon-β; IL-1β, interleukin-1β; IRF3, interferon regulatory factor 3; IRG1/ACOD1, immune-responsive gene 1/cis-aconitate decarboxylase; KEAP1, Kelch-like ECH-associated protein 1; MAMs, mitochondria-associated ER membranes; MFN1/2, mitofusin 1/2; mPTP, mitochondrial permeability transition pore; mtDNA, mitochondrial DNA; mtROS, mitochondrial reactive oxygen species; NAD⁺, nicotinamide adenine dinucleotide; NLRP3, NLR family pyrin domain containing 3; Nrf2, nuclear factor erythroid 2-related factor 2; OPA1, optic atrophy protein 1; PHD, prolyl hydroxylase; SIRT3, sirtuin 3; SOD2, superoxide dismutase 2; STING, stimulator of interferon genes; TBK1, TANK-binding kinase 1; TCA, tricarboxylic acid; Trx, thioredoxin; TXNIP, thioredoxin-interacting protein.

As established in Section 2, cardiovascular pathogenesis arises from feedback between metabolism and inflammation rather than from either process in isolation. Energetic intermediates—citrate, succinate, lactate, α-KG—act as direct mediators of inflammation onset and resolution, while cytokines reshape metabolism in return, producing both negative- and positive-feedback circuits. Figure 2 lays out four dimensions in which mitochondria translate metabolic stress into innate immunity: dynamics imbalance, mtDNA-driven cGAS–STING signaling, mtROS-mediated NLRP3 inflammasome assembly, and immunometabolite (succinate, itaconate, α-KG) signaling.

### 3.1 Mitochondrial dynamics imbalance: from fusion–fission to inflammatory activation

The mitochondrial network is in constant motion. Fusion and fission together maintain organelle morphology, function, and quality control, and the same remodeling cycle supports pluripotency, proliferation, differentiation, senescence, and cell death (60). Bioenergetics, calcium handling, and ROS control all rest on this balance, and in cardiovascular disease its disruption is an early event in metabolic reprogramming and inflammatory activation.

#### 3.1.1 Molecular regulation of fusion and fission

Outer-membrane fusion depends on the GTPases mitofusin 1 and 2 (MFN1/MFN2), which tether and merge adjacent membranes via GTP hydrolysis. Structural work on MFN1 indicates that nucleotide-induced GTPase-domain dimerization carries the reaction (61). On the inner membrane, optic atrophy protein 1 (OPA1) performs the equivalent function and also stabilizes cristae and gates cytochrome c release. Fission and fusion together regulate mitophagy in cardiomyocytes and fibroblasts (62); cardiac DRP1 inactivation produces hypertrophic mitochondria, dilated cardiomyopathy, and cell death, whereas combined cardiomyocyte deletion of MFN1/MFN2 causes fragmentation and pathological remodeling.

DRP1—the dynamin-related GTPase—is the principal fission effector, mediating mechanical scission within a multiprotein “fissome” assembled at ER–mitochondria contact sites. These contact sites (MAMs) couple the fission machinery to Ca^2+^ exchange and phospholipid transfer, giving metabolism and inflammation a shared structural interface. Lipid, Ca^2+^, and ROS flux through MAMs adjust bioenergetics and dictate survival under stress; MAMs also organize hormonal and nutrient signaling that affects insulin sensitivity and glucose homeostasis (63). OPA1 is processed into long (L-OPA1) and short (S-OPA1) forms by the YME1L/OMA1 protease pair, and this balance is required for adult cardiac mitochondrial homeostasis: cardiac *Yme1 L* ablation activates OMA1, accelerates OPA1 processing, and produces fragmented mitochondria with dilated cardiomyopathy and reduced contractility, whereas concurrent *Oma1* deletion blocks OPA1 cleavage and restores both morphology and cardiac function (64).

#### 3.1.2 Dynamics imbalance in cardiovascular disease

Across cardiac pathologies, fission–fusion balance is disrupted in one direction—excess fragmentation. Human atherosclerotic plaques show reduced mtDNA replication and lower oxygen consumption in the fibrous cap and core; smooth muscle cells from lesions have impaired bioenergetics, lower Complex I expression, and accelerated mitochondrial turnover, probably driven by oxidized LDL. *ApoE*-deficient mice overexpressing the Twinkle helicase have higher mtDNA copy number, raised respiratory chain expression, improved oxidative phosphorylation, smaller necrotic cores, and thicker fibrous caps (65). HF shows a parallel disruption: cardiac-specific double knockout of *Mfn1* and *Mfn2* causes severe fragmentation and metabolic dysfunction, with mutant cardiomyocytes showing biogenesis with empty matrices and damaged membranes, and the animals die before postnatal day 16 (66). Suppressing excess fission is correspondingly cardioprotective: in ischemia–reperfusion models, DRP1 inhibition improves diastolic relaxation and reduces fragmentation (67).

Mitophagy clears damaged mitochondria. The PINK1–Parkin axis has long been viewed as the main route (68, 69), but a more recent picture is more selective: PINK1–Parkin mitophagy is dispensable for basal cardiac homeostasis and only required under acute stress, while BNIP3/NIX-mediated receptor-dependent mitophagy dominates chronic adaptation (70). The therapeutic message is direct—non-selective mitophagy activators may do more harm than good in chronic HF, and stress-context-specific intervention is required.

### 3.2 mtDNA release and the cGAS–STING pathway

Mitochondria carry their own ∼16.6 kbp circular genome encoding core electron transport chain (ETC) subunits, 22 tRNAs, and 2 rRNAs. Unlike nuclear DNA, mtDNA is unprotected by histones and sits next to the principal cellular ROS source, leaving it highly vulnerable to oxidative damage. Under physiological conditions it is held within nucleoids; once mitochondria are damaged, it escapes into the cytosol and triggers inflammatory signaling (71).

#### 3.2.1 Routes of mtDNA release

mtDNA reaches the cytosol by several routes. The best-characterized is mitochondrial outer membrane permeabilization (MOMP), usually associated with programmed cell death: BAX and BAK oligomerize at the outer membrane and form macropores, and the inner membrane herniates through these pores to release its contents, including mtDNA (72). A second route opens through the mitochondrial permeability transition pore (mPTP), through which mtDNA can escape into the cytosol and activate cGAS–STING. Newly synthesized rather than oxidatively damaged mtDNA is selectively translocated to the cytosol and engages NLRP3: TLR signaling through MyD88 and TRIF induces IRF1-dependent upregulation of CMPK2, which supplies the dNTP pool required for mtDNA synthesis, and CMPK2-dependent mtDNA generation provides the oxidized fragments that activate the cytosolic NLRP3 inflammasome (73). Failure to clear mtDNA through autophagy promotes myocarditis and dilated cardiomyopathy via TLR9 signaling: in cardiac-specific DNase II-deficient mice, pressure overload causes mtDNA accumulation in autolysosomes, immune cell recruitment, cytokine release, myocarditis, and HF—all prevented by TLR9 inhibition (74).

#### 3.2.2 cGAS–STING signal transduction

cGAS is the cytosolic dsDNA sensor that initiates type I interferon (IFN) production (75). On binding cytosolic DNA, cGAS synthesizes 2′,3′-cGAMP, which activates STING (76). cGAMP binding reshapes the STING dimer and drives translocation from the endoplasmic reticulum through the ER–Golgi intermediate compartment to the Golgi, where STING recruits TBK1 and IKK. TBK1-mediated phosphorylation of MAVS, STING, and TRIF activates IRF3, with phosphorylated STING providing the docking site for IRF3 (77). cGAS–STING ultimately couples type I IFN production with NF-κB-driven inflammation.

#### 3.2.3 cGAS–STING in cardiovascular disease

Cytosolic DNA sensing during myocardial ischemia drives macrophage phenotypic switching and modifies ischemic injury (78). After myocardial infarction, excess IRF3 and type I IFN signaling provoke a fatal inflammatory response: genetic deletion of *Irf3* or *Ifnar* improves post-infarct cardiac function and survival in mice, identifying type I IFN signaling as deleterious in this setting (79). In atherosclerosis, cytosolic DNA and STING promote vascular smooth muscle cell proliferation and aortic wall degeneration and contribute to dissection and rupture; STING gene deletion or pharmacological inhibition limits these lesions (80). Cytosolic DNA also engages a STING-dependent cell-death program in myeloid cells that sits upstream of NLRP3, linking cGAS–STING to other innate immune circuits (81).

### 3.3 Mitochondrial ROS and the NLRP3 inflammasome

At low concentrations, ROS act as physiological signaling molecules regulating proliferation, differentiation, and stress responses. When ROS output exceeds antioxidant defense, the balance shifts toward oxidative damage of DNA, proteins, and lipids. Mitochondria are the principal cellular ROS source, providing a direct link between metabolism and inflammation.

#### 3.3.1 Generation and regulation of mtROS

The dominant ROS source is the ETC: ∼0.2%–2% of electrons leak from complexes I and III and react with O₂ to form superoxide (O₂•⁻) (82). Succinate accumulates in ischemic tissue, and on reperfusion it drives reverse electron flow through complex I to generate large amounts of mtROS—a primary mechanism of ischemia–reperfusion injury (IRI); pharmacological or genetic suppression of succinate accumulation attenuates IRI in animal models (2).

#### 3.3.2 mtROS as a second signal for NLRP3 activation

The NLRP3 inflammasome has three components—the sensor NLRP3, the adaptor ASC, and the effector pro-caspase-1—and assembles in two steps. A priming signal upregulates *NLRP3* and *pro–IL-1*β through TLR- or cytokine-receptor-driven NF-κB activation; an activation signal then triggers assembly in response to diverse DAMPs (83), mtROS act as that second signal—all canonical NLRP3 activators induce mtROS, and mitochondrially targeted antioxidants suppress NLRP3 activation (84). Cardiolipin, normally restricted to the inner mitochondrial membrane, is oxidized under stress and translocates to the outer membrane, where it binds the NLRP3 leucine-rich repeat (LRR) domain and is required for inflammasome assembly (85). NF-κB also restrains inflammasome activation by promoting clearance of damaged mitochondria; once fission becomes pathological, mitophagy fails and dysfunctional mitochondria activate NLRP3 persistently (86).

#### 3.3.3 NLRP3 inflammasome in cardiac and vascular disease

NLRP3 activation underlies sterile inflammation across cardiovascular conditions (87). In early atherogenesis, cholesterol crystals accumulate alongside leukocyte infiltration and activate NLRP3 through phagolysosomal disruption: bone marrow transplantation from NLRP3-, ASC-, or IL-1α/β-deficient mice into *Ldlr*⁻/⁻ recipients reduces early atherosclerosis on a high-cholesterol diet, identifying cholesterol crystals as endogenous DAMPs that drive—rather than passively accompany—inflammation (88). After acute myocardial infarction, the inflammasome detects tissue damage and amplifies inflammation through IL-1β and IL-18 release; the response is shared by ischemic and non-ischemic injury and drives adverse remodeling and HF progression, with early clinical studies of IL-1β blockade showing improved myocardial outcomes (89). Canakinumab acts on the cytokine output of the NLRP3 axis, while colchicine acts further upstream at tubulin-dependent inflammasome assembly and mitochondrial trafficking; CANTOS, COLCOT, and LoDoCo2 jointly demonstrate that the NLRP3–IL-1β branch can be inhibited clinically at multiple nodes (8–10). MCC950, a selective small-molecule NLRP3 inhibitor, binds the NLRP3 ATP-hydrolysis motif and blocks inflammasome assembly (90), and in a porcine MI model it reduces infarct size and preserves cardiac function (91).

### 3.4 Inflammatory regulation by mitochondrial metabolites

Beyond mtDNA and mtROS, mitochondria modulate immunity through metabolic signaling molecules. Succinate, fumarate, itaconate, and α-KG act as immunometabolic mediators regulating transcription, epigenetics, and post-translational modifications.

#### 3.4.1 Succinate

Succinate promotes M1 polarization through both intracellular and receptor-mediated mechanisms. SDH inhibition by dimethyl malonate is anti-inflammatory (23); inside the cell, the succinate–PHD–HIF-1α–IL-1β circuit (Section 2.2.1) is reinforced by SDH-derived mtROS. Succinate also acts extracellularly through SUCNR1 (GPR91): myeloid-specific *Sucnr1* deletion in mice produces a pro-inflammatory phenotype, disrupts glucose homeostasis on a chow diet, worsens obesity-driven metabolic disease, and impairs cold-induced beiging of white adipose tissue, whereas SUCNR1 agonism shifts macrophages toward an anti-inflammatory state and enhances type 2 cytokine sensitivity (92).

#### 3.4.2 Itaconate

Itaconate alkylates KEAP1 cysteine residues (Cys151, Cys257, Cys273, Cys288, Cys297) and releases Nrf2 to drive antioxidant and anti-inflammatory gene expression, with Nrf2 activation required for the anti-inflammatory effect (5). Itaconate also directly suppresses NLRP3: itaconate and 4-OI inhibit NLRP3 but not AIM2 or NLRC4, acting through dicarboxypropylation of NLRP3 Cys548 that disrupts the NLRP3–NEK7 interaction needed for inflammasome assembly (93).

#### 3.4.3 Fumarate

Fumarate succinates essential cysteine residues on gasdermin D, blocks proteolytic activation, and prevents pyroptotic pore formation (94). The clinical immunomodulator dimethyl fumarate targets GAPDH and aerobic glycolysis, dampening pro-inflammatory signaling through glycolytic restraint (95).

#### 3.4.4 NAD^+^ and sirtuins

NAD⁺ is a fundamental redox coenzyme, and the NAD⁺-dependent sirtuin family (SIRT1–SIRT7) constitutes one metabolite-sensing arm of the metabolic–epigenetic axis relevant to cardiovascular disease (96). SIRT3 is the main mitochondrial deacetylase and regulates FAO through reversible protein deacetylation (97); it also deacetylates SOD2 at K68/K122 to enhance mitochondrial antioxidant defense (98), *Sirt3*⁻/⁻ mice develop cardiac hypertrophy and interstitial fibrosis with FOXO3a-dependent loss of MnSOD/catalase induction, while cardiac SIRT3 transgenic mice are protected from hypertrophic stimuli (99). Cardiac SIRT3 expression is reduced in failing human hearts and in multiple animal models, which has prompted its proposal as a candidate biomarker and therapeutic target in HF; prospective validation in adequately powered cardiovascular cohorts remains pending. SIRT1, the nuclear paralog, deacetylates p65, FOXO1/3, and PGC-1α, linking caloric-restriction signals to cardiomyocyte and endothelial homeostasis, and endogenous SIRT1 is consistently cardioprotective in cardiac stress and aging models. SIRT6, a chromatin-bound NAD⁺-dependent deacetylase, restrains pathological hypertrophy by suppressing IGF–Akt signaling at c-Jun target chromatin; SIRT6 expression is reduced in failing human hearts, *SIRT6*⁻/⁻ mice develop hypertrophy and HF, and cardiac-specific SIRT6 transgenic mice are protected from hypertrophic stimuli (100). NAD⁺-boosting compounds (NMN, NR) thus have broad therapeutic potential, with NMN supplementation reversing aging-related vascular dysfunction in mice (101) and shifting vascular miRNA profiles in aged mice toward an anti-aging signature consistent with epigenetic rejuvenation and atheroprotection (102).

One question remains open: if a single metabolite—succinate—powers both mtROS-induced NLRP3 activation and HIF-1α-mediated transcription, can compartment-selective interventions (mitochondrial vs. cytosolic succinate buffering) separate the inflammatory from the epigenetic output in animal models of cardiovascular disease?

## 4 The metabolic–epigenetic axis: metabolic control of inflammatory gene expression

Metabolites are more than permissive cofactors for epigenetic enzymes—in cardiovascular cells they set the pace of inflammatory gene expression. Acetyl-CoA controls p300/CBP-mediated H3K27ac at pro-inflammatory loci, α-ketoglutarate tunes demethylase activity through JMJD3 and TET2, and lactate runs a “lactate clock” through histone lactylation that times the resolution of inflammation. The consequence is that brief metabolic perturbations leave durable, heritable inflammatory phenotypes—the mechanism by which trained immunity takes hold in cardiovascular disease.

Epigenetic modifications—stable, heritable changes in gene expression that do not alter nucleotide sequence—include DNA methylation, histone post-translational modifications, and chromatin remodeling. Cellular metabolic state shapes these modifications directly by controlling the supply of metabolite substrates that chromatin-modifying enzymes require. In cardiovascular disease, this coupling turns a transient metabolic insult into chronic inflammation and immunological memory (103).

### 4.1 Acetyl-CoA and histone acetylation: the epigenetic basis of trained immunity

#### 4.1.1 Acetyl-CoA compartmentalization

Acetyl-CoA sits at the crossroads of central metabolism and serves as the required acetyl donor for histone acetyltransferases (HATs). Its mitochondrial, cytosolic, and nuclear pools are largely separate, each fed by distinct routes: the dominant nuclear-cytoplasmic source is ACLY (introduced in Section 2.2.1), which cleaves mitochondrion-derived citrate into acetyl-CoA and oxaloacetate, while acetyl-CoA synthetase 2 (ACSS2) supplies a parallel route by ligating free acetate to coenzyme A. Nuclear acetyl-CoA concentration tracks nutrient availability and rises with global histone acetylation.

#### 4.1.2 ACLY-dependent histone acetylation and inflammatory genes

**Figure 3:**
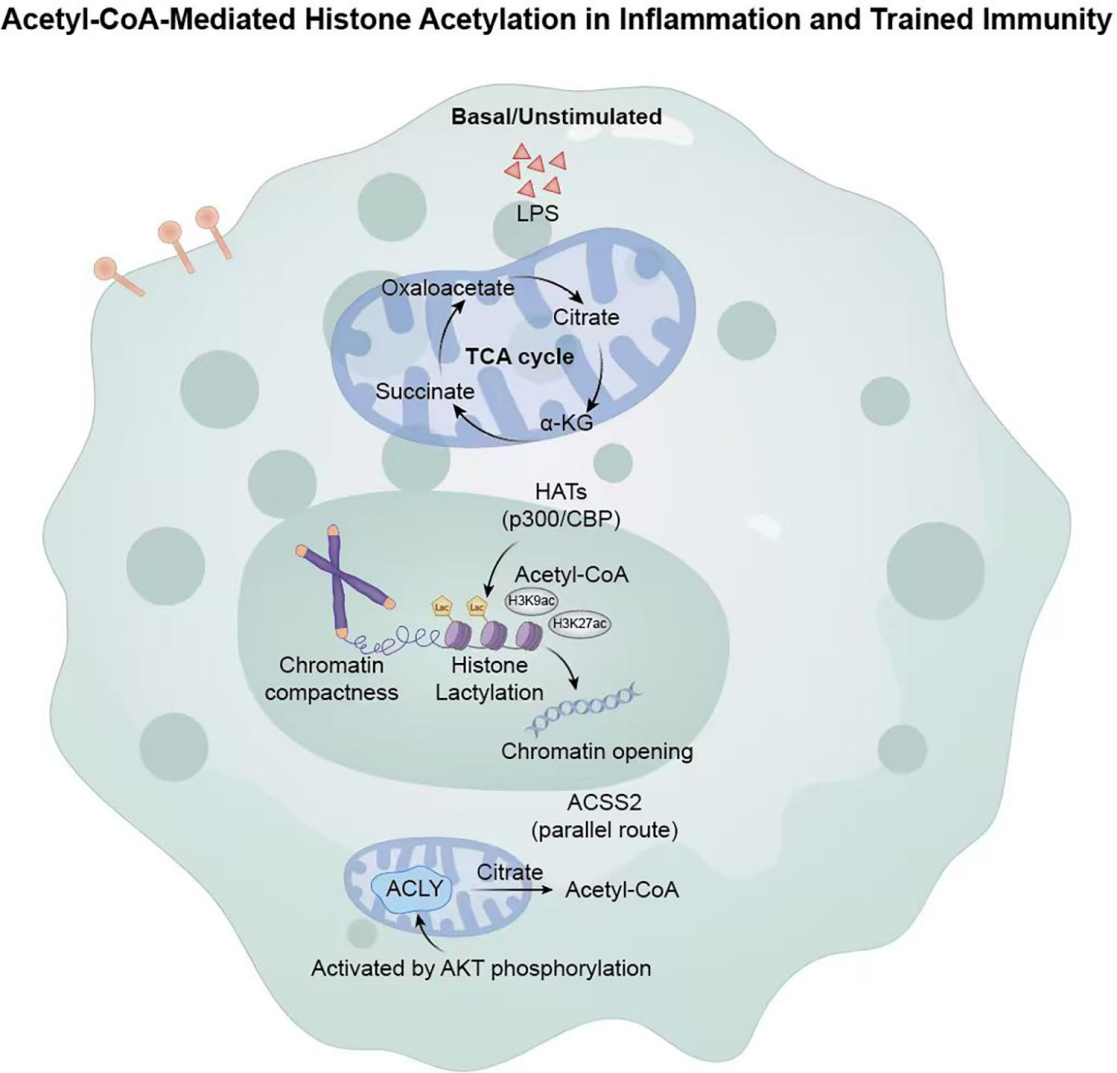
ACLY-dependent acetyl-CoA generation drives histone acetylation and chromatin opening at pro-inflammatory loci upon TLR engagement. In resting macrophages, the TCA cycle operates intact and chromatin remains in a compact, transcriptionally restrictive state. Following LPS recognition by TLR4, MyD88/TRIF-dependent signaling triggers AKT-mediated phosphorylation and activation of cytosolic ATP-citrate lyase (ACLY). Mitochondrion-derived citrate is exported to the cytosol, where ACLY cleaves it into acetyl-CoA and oxaloacetate; acetyl-CoA synthetase 2 (ACSS2) provides a parallel, complementary route by ligating free acetate to coenzyme A. The resulting nuclear acetyl-CoA pool serves as the obligate acetyl donor for p300/CBP histone acetyltransferases (HATs), which selectively deposit H3K9ac and H3K27ac at pro-inflammatory promoters such as *Il6* and *Il12b*. Glycolysis-derived lactate concurrently drives p300-catalyzed histone lysine lactylation (Kla) at competing lysine residues, establishing a parallel chromatin mark. The integrated effect is a transition from compact chromatin to an open conformation that is permissive to transcription factor recruitment and inflammatory gene activation. ACLY, ATP-citrate lyase; ACSS2, acetyl-CoA synthetase 2; α-KG, α-ketoglutarate; HATs, histone acetyltransferases; Kla, histone lysine lactylation; LPS, lipopolysaccharide; TCA, tricarboxylic acid; TLR, toll-like receptor.

Histone acetylation neutralizes the lysine positive charge, weakens the histone–DNA contact, and opens chromatin to transcription factors, with the HAT–HDAC balance setting this state. The TCA-cycle interruption described in Section 2.2.1—IDH inhibition with citrate accumulation and SDH inhibition with succinate accumulation—provides cytosolic citrate that ACLY converts to acetyl-CoA. TLR signaling rewires this flux: ¹³C-glucose tracing showed that LPS-induced, glucose-derived acetyl-CoA selectively raises H3 acetylation at K9, K14, K18, K23, and K27, with H3K27ac enriched at the *Il6* and *Il12b* promoters; the response depends on MyD88/TRIF-mediated ACLY phosphorylation, and ACLY inhibition with BMS 303141 suppresses *Il6* and *Il12b* without affecting primary-response genes (104). p300/CBP is the main HAT for inflammatory genes: NF-κB-driven recruitment of p300/CBP acetylates local histones and also acetylates p65 at Lys310, strengthening p65 transactivation and stability (105). Figure 3 summarizes this circuit, from TLR-triggered ACLY activation and citrate-derived acetyl-CoA flux to p300/CBP-mediated H3K9ac and H3K27ac at inflammatory promoters.

#### 4.1.3 Trained immunity as metabolic–epigenetic memory

**Figure 4:**
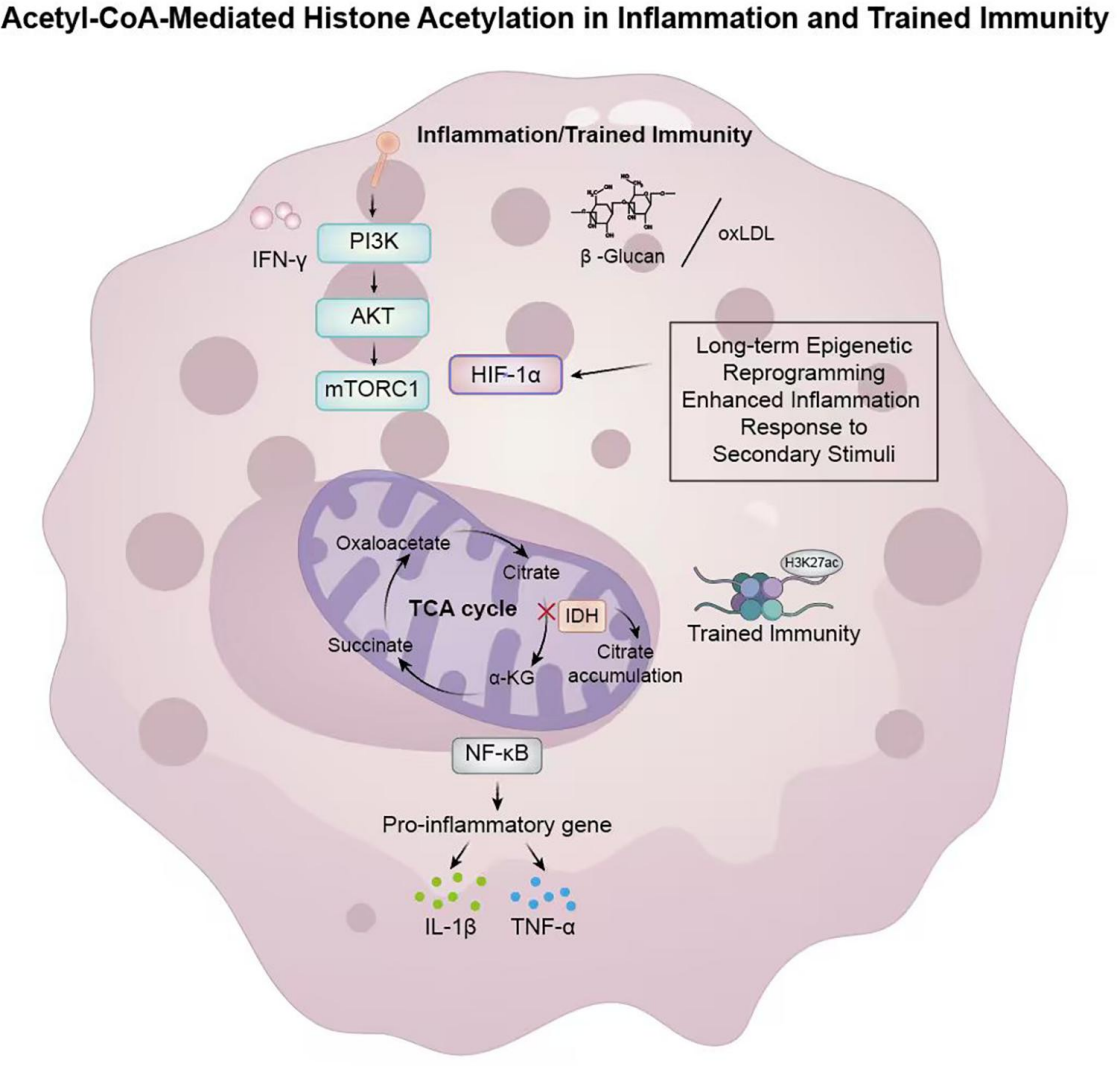
Sustained metabolic–epigenetic reprogramming establishes trained immunity in cardiovascular inflammation. Engagement of innate pattern-recognition receptors by β-glucan or oxidized LDL (oxLDL), together with priming cytokines such as IFN-γ, activates the PI3K–AKT–mTORC1 signaling axis and stabilizes hypoxia-inducible factor-1α (HIF-1α). This drives a sustained glycolytic switch and remodeling of the TCA cycle: isocitrate dehydrogenase (IDH) activity declines (red cross), producing chronic citrate accumulation, while succinate also accumulates downstream. The persistent metabolic state continuously feeds ACLY-dependent acetyl-CoA generation and supports durable enrichment of H3K4me3 and H3K27ac at enhancers of pro-inflammatory genes — encoding a long-term epigenetic memory (“trained immunity”). Upon secondary stimulation, the primed chromatin landscape licenses amplified NF-κB recruitment and an exaggerated IL-1β and TNF-α response, providing a molecular basis for the persistent myeloid hyper-responsiveness observed in atherosclerotic cardiovascular disease. HIF-1α, hypoxia-inducible factor-1α; IDH, isocitrate dehydrogenase; IFN-γ, interferon-γ; mTORC1, mechanistic target of rapamycin complex 1; NF-κB, nuclear factor-κB; oxLDL, oxidized low-density lipoprotein; PI3K, phosphoinositide 3-kinase; TCA, tricarboxylic acid; TNF-α, tumor necrosis factor-α.

In trained immunity, β-glucan signaling through Dectin-1 switches metabolism toward glycolysis and glutaminolysis under mTOR–HIF-1α control; the resulting acetyl-CoA flux supports H3K4me3 and H3K27ac at inflammatory-gene enhancers, and blocking glycolysis, mTOR, or ACLY abolishes the trained phenotype (106). Figure 4 shows how sustained PI3K–AKT–mTORC1–HIF-1α signaling, triggered by β-glucan, oxLDL, or IFN-γ, maintains TCA-cycle remodeling and citrate accumulation, imprints durable H3K4me3 and H3K27ac marks, and licenses an amplified IL-1β/TNF-α response on secondary challenge. Trained immunity is now an accepted mechanism in atherosclerotic disease, demonstrated directly in symptomatic and asymptomatic human atherosclerosis (107). OxLDL drives a monocyte chromatin state that resembles β-glucan training and confers lasting hyper-responsiveness (108). In *Ldlr*⁻/⁻ mice, a Western diet drove systemic inflammation that resolved on dietary reversal, yet myeloid cells remained hyper-responsive to PAMPs through persistent epigenetic reprogramming of myeloid progenitors; *Ldlr*⁻/⁻*Nlrp3*⁻/⁻ double-knockout mice did not develop these changes, placing NLRP3 as the required sensor of diet-induced innate memory (109).

### 4.2 α-Ketoglutarate and dioxygenases: epigenetic control of macrophage polarization

α-Ketoglutarate is the co-substrate for more than 60 Fe^2+^/α-KG-dependent dioxygenases involved in oxygen sensing, DNA demethylation, and histone demethylation. Three subclasses matter most here: histone lysine demethylases (KDMs); the TET DNA dioxygenases, which oxidize 5-methylcytosine stepwise to 5hmC, 5fC, and 5caC; and prolyl hydroxylases, which target HIF-α for degradation. Their *K*ₘ values lie close to physiological intracellular α-KG, so small shifts in α-KG change their activity, and succinate, fumarate, and 2-hydroxyglutarate inhibit them competitively (110). Two enzymes are central to cardiovascular inflammation: JMJD3 (KDM6B) on the histone arm and TET2 on the DNA arm. The cellular α-KG/succinate ratio sets activity at both, coupling histone and DNA demethylation to the TCA cycle.

On the histone arm, glutaminolysis-derived α-KG powers JMJD3-mediated H3K27me3 erasure at the M2 signature genes *Arg1*, *Fizz1*, *Ym1*, and *Mrc1* while sustaining fatty-acid oxidation. The α-KG/succinate ratio works as a switch: high ratios favor M2 polarization while low ratios reinforce M1, and α-KG is also required for endotoxin tolerance; cell-permeable α-KG analogs are sufficient to drive M2 polarization in mice, whereas glutaminolysis blockade or JMJD3 loss prevents it (29). Further upstream, SENP1–SIRT3 signaling controls glutaminolysis flux through GLUD1 deacetylation, offering a regulatable handle on α-KG supply (111).

The DNA arm follows the same logic in vascular cells. TET dioxygenases use α-KG to oxidize 5mC and are competitively inhibited by the same metabolites that act on KDMs. In atheroprone arterial regions, disturbed hemodynamics suppress TET2 and hypermethylate the *KLF2* promoter in mouse and human endothelium, weakening endothelial homeostasis (112). TET2 loss-of-function mutations are also the most frequent driver of clonal hematopoiesis of indeterminate potential (CHIP), now an independent cardiovascular risk factor in human cohorts (113). In *Ldlr*⁻/⁻ mice, even partial reconstitution with TET2-mutant bone marrow produces clonal expansion, larger plaques, and raised NLRP3-mediated IL-1β secretion, and MCC950 gives greater plaque protection in TET2-deficient chimeras than in controls—tying age-associated clonal hematopoiesis to NLRP3-driven cardiovascular inflammation (114).

**Figure 5:**
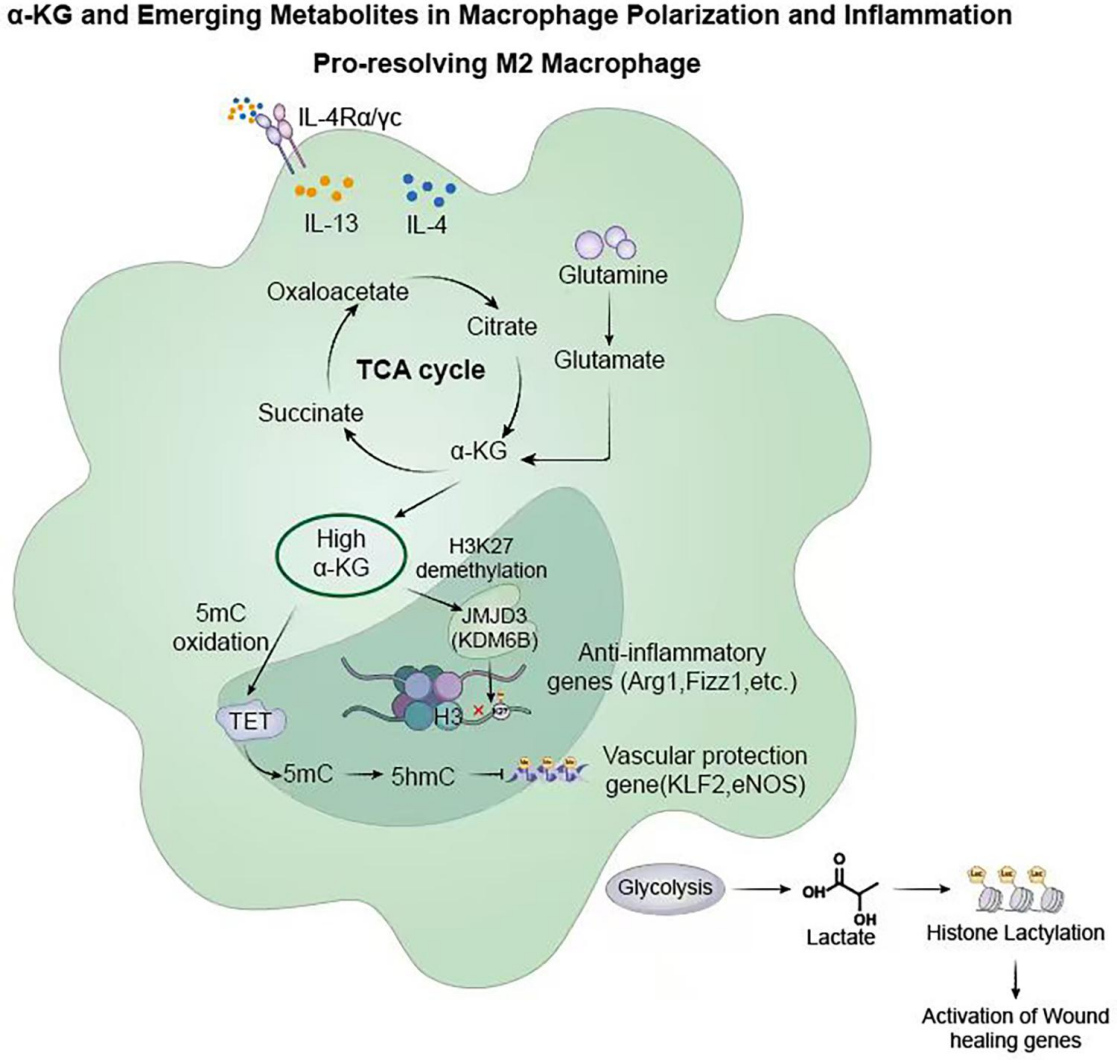
Glutaminolysis-derived α-ketoglutarate and emerging metabolites coordinate the epigenetic program of M2 polarization in pro-resolving macrophages. In response to IL-4 and IL-13, macrophages upregulate glutaminolysis: glutamine is converted to glutamate and subsequently to α-ketoglutarate (α-KG), replenishing the TCA cycle through anaplerosis and elevating intracellular α-KG levels. As the obligate co-substrate of Fe^2+^/α-KG-dependent dioxygenases, α-KG sustains two parallel chromatin-modifying arms. On the histone arm, the H3K27 demethylase JMJD3 (KDM6B) erases repressive H3K27me3 marks at M2 signature loci (*Arg1*, *Fizz1*), licensing anti-inflammatory gene transcription. On the DNA arm, TET dioxygenases oxidize 5-methylcytosine (5mC) to 5-hydroxymethylcytosine (5hmC) at vascular protective gene promoters (*KLF2*, *eNOS*), counteracting DNA hypermethylation and supporting endothelial homeostasis. In parallel, glycolysis-derived lactate fuels p300-catalyzed histone lysine lactylation (Kla), establishing a “lactate clock” that times the late-phase activation of wound-healing and tissue-repair genes and drives the inflammation-to-resolution transition. α-KG, α-ketoglutarate; eNOS, endothelial nitric oxide synthase; JMJD3 (KDM6B), Jumonji domain-containing protein 3; Kla, histone lysine lactylation; KLF2, Krüppel-like factor 2; TCA, tricarboxylic acid; TET, ten-eleven translocation dioxygenase.

The two arms meet on one circuit. In M1 macrophages, succinate stabilizes HIF-1α to drive IL-1β transcription (Section 2.2.1) while simultaneously inhibiting JMJD3 and TET2 at inflammatory promoters; TET2 deficiency in CHIP then amplifies the same NLRP3–IL-1β output downstream. Restoring cellular α-KG is the shared therapeutic node. Figure 5 summarizes this M2 program: IL-4/IL-13-driven glutaminolysis feeding α-KG supply, JMJD3-mediated H3K27me3 erasure at M2 loci (*Arg1*, *Fizz1*, *Ym1*, *Mrc1*), TET-driven 5mC oxidation at vascular protective genes (*KLF2*, *eNOS*), and glycolysis-derived lactate fueling the histone lactylation that times wound-healing gene activation.

### 4.3 Epigenetic regulation by emerging metabolites

#### 4.3.1 β-OHB as an endogenous HDAC inhibitor

**Figure 6:**
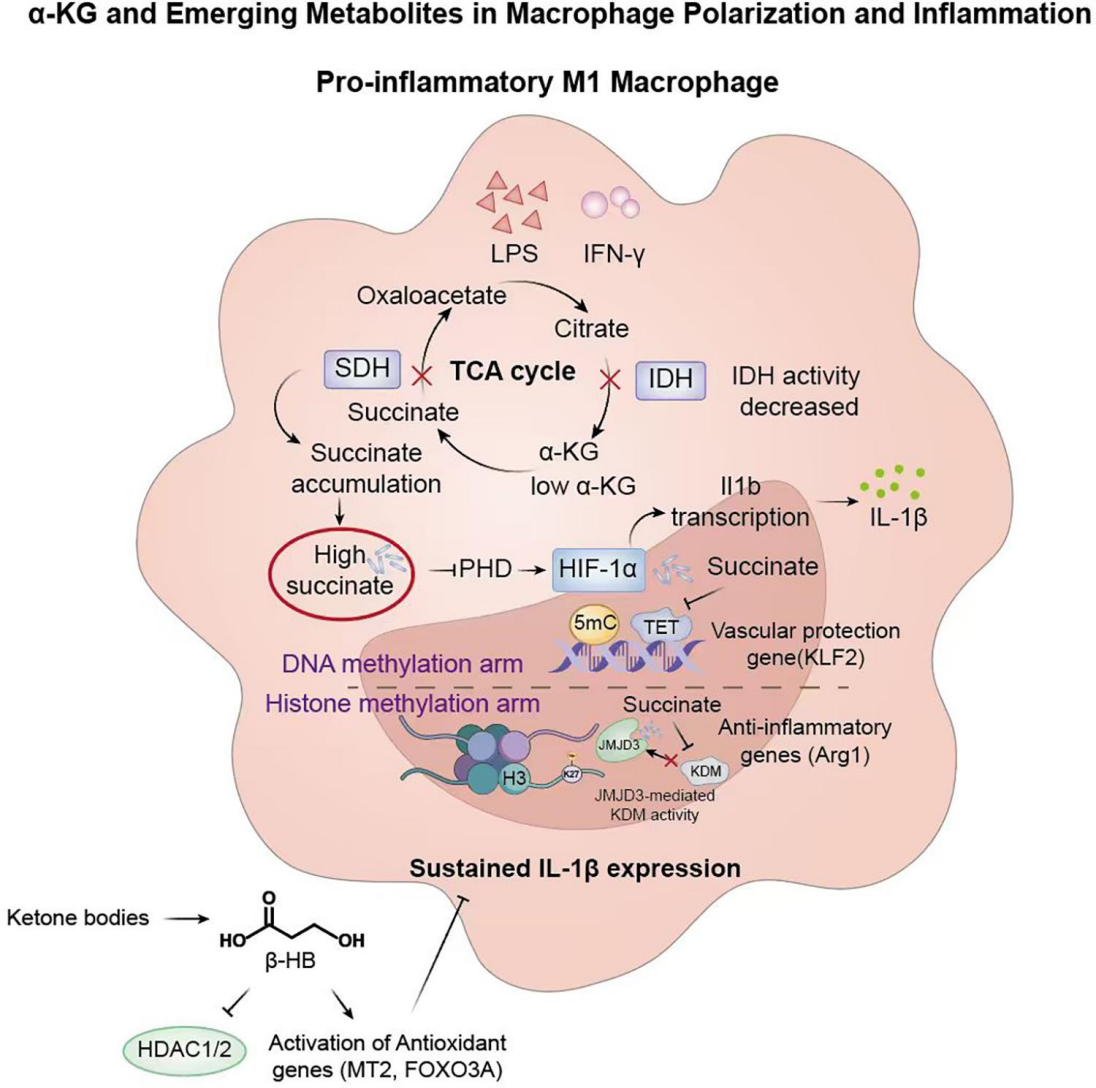
Succinate accumulation imposes a self-reinforcing pro-inflammatory epigenetic state in M1 macrophages, counter-regulated by β-hydroxybutyrate as an endogenous HDAC inhibitor. Upon stimulation with LPS and IFN-γ, the macrophage TCA cycle is interrupted at two nodes: isocitrate dehydrogenase (IDH) and succinate dehydrogenase (SDH) activities decline, producing intracellular succinate accumulation and α-ketoglutarate (α-KG) depletion. Succinate exerts dual epigenetic and signaling effects. First, it competitively inhibits prolyl hydroxylase (PHD), stabilizing HIF-1α and driving sustained transcription of pro-inflammatory genes including *Il1b*. Second, it competitively inhibits Fe^2+^/α-KG-dependent dioxygenases at chromatin: TET-mediated demethylation of vascular protective gene (*KLF2*) promoters is blocked, locking these loci in a hypermethylated, transcriptionally silenced state; concurrently, JMJD3/KDM-mediated H3K27me3 removal at M2 anti-inflammatory genes (*Arg1*) is suppressed, preventing the resolution program. The net result is sustained high IL-1β output. As a homeostatic counter-regulator, ketone-body-derived β-hydroxybutyrate (β-OHB) acts as an endogenous class-I HDAC1/2 inhibitor, elevating histone acetylation at oxidative stress response and antioxidant defense genes (*Foxo3a*, *Mt2*), thereby restraining the pro-inflammatory state. α-KG, α-ketoglutarate; β-OHB, β-hydroxybutyrate; FOXO3A, forkhead box O3A; HDAC, histone deacetylase; HIF-1α, hypoxia-inducible factor-1α; IDH, isocitrate dehydrogenase; JMJD3, Jumonji domain-containing protein 3; KDM, lysine demethylase; KLF2, Krüppel-like factor 2; MT2, metallothionein-2; PHD, prolyl hydroxylase; SDH, succinate dehydrogenase; TET, ten-eleven translocation dioxygenase.

β-Hydroxybutyrate (β-OHB) is the principal ketone body and an endogenous class-I HDAC inhibitor. Exogenous β-OHB, fasting, or caloric restriction raise circulating β-OHB and global histone acetylation, with selective induction of oxidative-stress-response genes including *FOXO3A* and *MT2*, and β-OHB-treated mice are protected from oxidative injury (115). In cardiovascular disease, much of the benefit of ketone-body use reflects this transcriptional reprogramming: β-OHB raises H3K9ac and H3K14ac through HDAC1/2 inhibition, also inhibits the NLRP3 inflammasome, and is cardioprotective in mouse ischemia–reperfusion and HF models (116). Figure 6 places this against the M1 state: TCA-cycle interruption and succinate accumulation stabilize HIF-1α, block TET-mediated demethylation at protective loci (*KLF2*), and inhibit JMJD3-mediated H3K27me3 erasure at M2 genes (*Arg1*), while β-OHB acts as a class-I HDAC1/2 inhibitor that turns on antioxidant transcription (*FOXO3A*, *MT2*).

#### 4.3.2 Lactate and histone lactylation

Histone lysine lactylation (Kla) is a chromatin mark deposited from glycolysis-derived lactate, with 28 sites mapped on core histones. In bacteria-stimulated M1 macrophages, lactylation peaks later than acetylation; late-phase M1 macrophages carry high H3K18la at homeostatic and tissue-repair genes such as *Arg1*, supporting an intrinsic “lactate clock” that times inflammation resolution (13). Lactylation is now firmly part of cardiovascular biology: after myocardial infarction, H3K18la rises in circulating monocytes before cardiac recruitment and activates *Lrg1*, *Vegfa*, and *Il10*, setting up the reparative monocyte program before the cells reach the infarct (117). The TLR adaptor BCAP likewise pushes the inflammatory-to-reparatory transition forward by raising histone lactylation (118).

The enzymatic machinery runs in both directions. p300 deposits the lactyl mark, and HDAC1, HDAC2, and HDAC3—long classed as deacetylases—also act as histone delactylases (119). Lactylation and acetylation thus share writers and erasers, and the two marks compete for the same metabolite pool and enzyme capacity. Whether histone lactylation alone is sufficient to drive the M1-to-M2 transition, or whether it is one of several parallel marks needed for full reprogramming, remains an open question; the cardiovascular replication discussed in Section 6.5(iii) is therefore framed as genetic uncoupling that argues for causality, not as proof.

Lactylation, acetylation, and methylation draw on competing metabolite pools. What sets the order in which these marks are written and erased during the M1-to-M2 transition, and can that order be rewritten therapeutically?

## 5 Lipid peroxidation and ferroptosis as a readout of the metabolic–epigenetic axis

We take the position that ferroptosis in cardiovascular disease is not a separate injury pathway sitting alongside metabolic and inflammatory dysfunction—it is what the metabolic–epigenetic axis discussed in Sections 3 and 4 looks like once homeostatic capacity is exhausted. Three points support this view. First, the canonical ferroptotic machinery (SLC7A11/GSH/GPX4, ACSL4-driven PUFA-PE remodeling, NCOA4-mediated ferritinophagy) is governed by transcription factors—NRF2, HIF-1α, and p53—whose activity is set by the same TCA-cycle metabolites discussed earlier (succinate, itaconate, α-ketoglutarate). Second, the genes encoding this machinery (*GPX4*, *ACSL4*, *ALOX15*) are themselves regulated by DNA, RNA, and histone modifications whose substrates are metabolites. Third, arachidonic acid metabolism—long studied as a parallel inflammatory system—is routed between resolution and ferroptotic death by the same chromatin marks that regulate cytokine genes. Ferroptosis thus reads out the metabolic–epigenetic state of the cell.

**Figure 7:**
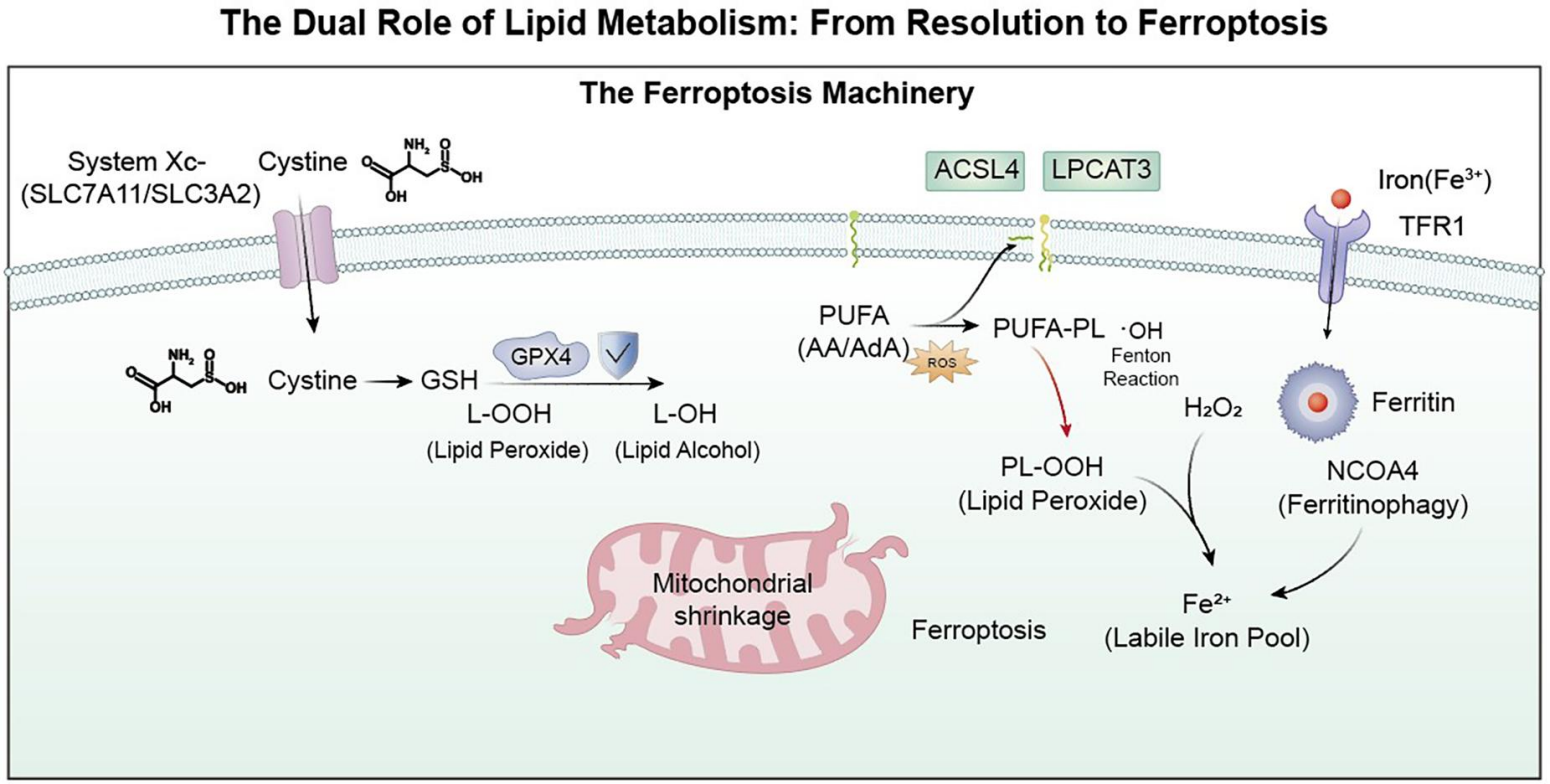
The core molecular machinery of ferroptosis: the SLC7A11/GSH/GPX4 antioxidant axis, ACSL4-driven phospholipid remodeling, and ferritinophagy-released labile iron converge on lipid peroxide accumulation. Ferroptosis is executed through three convergent molecular arms. (1) The cystine–GSH–GPX4 antioxidant arm: the system Xc⁻ antiporter (SLC7A11/SLC3A2) imports cystine in exchange for glutamate; intracellular cystine is reduced to cysteine, the rate-limiting precursor of glutathione (GSH). GSH supplies glutathione peroxidase 4 (GPX4), which reduces toxic phospholipid hydroperoxides (L-OOH) to non-toxic phospholipid alcohols (L-OH), terminating the lipid peroxidation chain. (2) The ACSL4–LPCAT3 membrane-remodeling arm: long-chain acyl-CoA synthetase 4 (ACSL4) and lysophosphatidylcholine acyltransferase 3 (LPCAT3) sequentially activate and incorporate polyunsaturated fatty acids (PUFAs)—particularly arachidonic acid (AA) and adrenic acid (AdA)—into membrane phospholipids (PUFA-PL), generating the obligate substrate pool for peroxidation. (3) The TfR1–ferritinophagy iron-loading arm: transferrin receptor 1 (TfR1) imports Fe^3+^, which is stored within ferritin and selectively released as labile Fe^2+^ through NCOA4-mediated ferritinophagy. The released labile iron drives Fenton chemistry (Fe^2+^ + H_2_O_2_ → ·OH), generating hydroxyl radicals that oxidize PUFA-PL into PL-OOH. When GPX4 capacity is exceeded, accumulating PL-OOH propagates an iron-dependent peroxidation cascade, culminating in mitochondrial shrinkage with cristae loss and ferroptotic cell death. AA, arachidonic acid; AdA, adrenic acid; ACSL4, acyl-CoA synthetase long-chain family member 4; GPX4, glutathione peroxidase 4; GSH, glutathione; LPCAT3, lysophosphatidylcholine acyltransferase 3; NCOA4, nuclear receptor coactivator 4; PUFA, polyunsaturated fatty acid; PUFA-PL, PUFA-containing phospholipid; PL-OOH, phospholipid hydroperoxide; ROS, reactive oxygen species; SLC3A2/SLC7A11, system Xc⁻ antiporter subunits; TfR1, transferrin receptor 1.

**Figure 8:**
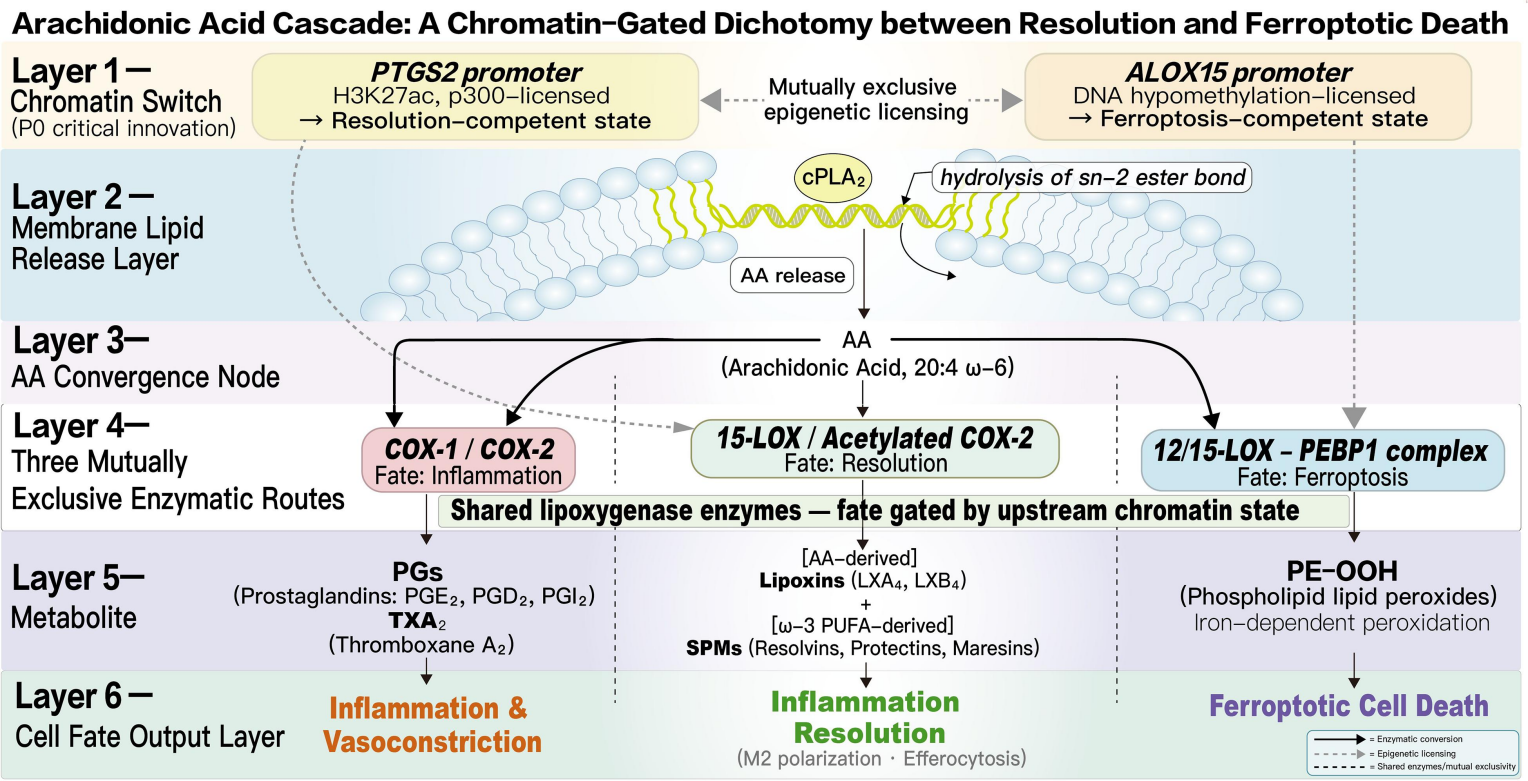
The arachidonic acid (AA) cascade is routed between three mutually exclusive enzymatic outputs — prostanoid signaling, pro-resolving mediator generation, and ferroptotic execution — by enzyme-substrate channeling, with branch selection gated upstream by chromatin state. AA, liberated from membrane phospholipids by cytosolic phospholipase A_2_ (cPLA_2_) through hydrolysis of the sn-2 ester bond, enters three downstream routes. (1) The cyclooxygenase (COX-1/2) route generates prostaglandins (PGs: PGE_2_, PGD_2_, PGI_2_) and thromboxane A_2_ (TXA_2_), driving inflammation and vasoconstriction. (2) The 15-LOX/aspirin-acetylated COX-2 route generates AA-derived lipoxins (LXA_4_, LXB_4_) and ω-3 PUFA-derived specialized pro-resolving mediators (SPMs: resolvins, protectins, maresins), which drive macrophage M2 polarization and efferocytosis to orchestrate inflammation resolution. (3) The 12/15-LOX–PEBP1 complex shifts lipoxygenase substrate preference from free PUFAs to membrane phosphatidylethanolamines, generating hydroperoxy-PE death signals (PE-OOH) that, when GPX4 detoxification is overwhelmed, drive iron-dependent ferroptotic execution. Importantly, routes (2) and (3) share the same lipoxygenase enzymes; the resolution-vs.-death dichotomy is therefore determined upstream by the chromatin state at *PTGS2* (encoding COX-2, p300/H3K27ac-licensed for resolution-competent flux toward acetylated COX-2 and lipoxin synthesis) vs. *ALOX15* (encoding 15-LOX, DNA hypomethylation-licensed for ferroptosis-competent flux toward PEBP1-directed PE peroxidation). The same arachidonic acid cascade therefore terminates in either tissue repair or iron-dependent membrane destruction depending on the upstream metabolic–epigenetic state. AA, arachidonic acid; *ALOX15*, arachidonate 15-lipoxygenase gene; COX-1/2, cyclooxygenase-1/2; cPLA_2_, cytosolic phospholipase A₂; GPX4, glutathione peroxidase 4; LOX, lipoxygenase; PE, phosphatidylethanolamine; PEBP1, phosphatidylethanolamine-binding protein 1; PGs, prostaglandins; *PTGS2*, prostaglandin-endoperoxide synthase 2 gene; PUFA, polyunsaturated fatty acid; SPMs, specialized pro-resolving mediators; TXA_2_, thromboxane A_2_.

Ferroptosis, an iron-dependent regulated cell death driven by uncontrolled lipid peroxidation, has been documented in atherosclerosis, acute coronary syndromes, and cardiac dysfunction (120–122). This section approaches the dual role of lipid metabolism from two angles. Figure 7 lays out the core molecular machinery of ferroptotic execution. Figure 8 shows how the arachidonic acid cascade is routed between resolution and ferroptotic outputs by enzyme–substrate channeling.

### 5.1 Molecular mechanisms of ferroptosis

#### 5.1.1 Defining features and the Cystine–GSH–GPX4 axis

Erastin triggers ferroptosis by blocking cystine import through system xc⁻ and collapsing cellular antioxidant capacity, and ferrostatin-1 blocks the process (123). The defining features are iron dependence, lipid ROS accumulation, mitochondrial shrinkage with cristae loss, and caspase-independence (122, 124). The xc⁻ antiporter, formed by SLC7A11 and SLC3A2, imports cystine in exchange for glutamate (125). Cystine is reduced inside the cell to cysteine, the limiting precursor of GSH; GSH then supplies GPX4, the enzyme that reduces lipid hydroperoxides to alcohols and terminates the peroxidation chain (126, 127). Conditional *Gpx4* deletion produces acute renal failure in mice (126), and loss of GPX4 activity or GSH depletion sets off the lipid peroxidation cascade that defines ferroptosis (128). Inhibiting xc⁻ depletes GSH, disables GPX4, and triggers ferroptosis (129).

#### 5.1.2 ACSL4-dependent membrane remodeling

PUFAs (polyunsaturated fatty acids) are required substrates for ferroptosis (130). Redox lipidomics shows that oxidation selectively targets ER-localized phosphatidylethanolamines (PEs) enriched in arachidonic acid (AA) and adrenic acid (AdA), and blocking AA/AdA incorporation into PE through ACSL4 silencing or pharmacological inhibition prevents ferroptosis (130, 131). ACSL4 and LPCAT3 carry out the incorporation, and the resulting PE species are oxidized by Fenton chemistry or lipoxygenase activity (131, 132). 15-Lipoxygenases generate the hydroperoxy-PE death signals, and tocopherols inhibit this step by suppressing LOX activity.

#### 5.1.3 Iron loading and ferritinophagy

Labile iron drives ferroptosis catalytically. Iron enters cells through transferrin receptor 1 and is stored in ferritin (133); NCOA4 is the selective autophagy receptor that delivers ferritin to lysosomes, releasing Fe^2+^ (134), and the released iron drives Fenton chemistry, producing hydroxyl radicals that propagate lipid peroxidation (135). Figure 7 brings the three arms together: the cystine–GSH–GPX4 antioxidant arm, ACSL4/LPCAT3-mediated incorporation of PUFAs into membrane phospholipids, and TfR1/NCOA4-driven labile-iron generation that feeds Fenton chemistry.

#### 5.1.4 Epigenetic control of the ferroptosis machinery

The genes that encode the ferroptotic machinery are themselves regulated by metabolite-dependent epigenetic enzymes. Three layers operate. First, DNA methylation. Under sustained oxidative stress, DNMT-mediated CpG hypermethylation of the *GPX4* promoter silences *GPX4* transcription, while α-KG-dependent TET activity opposes this silencing—linking *GPX4* transcriptional capacity directly to the cellular succinate/α-KG ratio. In the cardiovascular system, α-KG supplementation raises TET2 activity in vascular smooth muscle cells and protects against vascular calcification in mice (136). A cell with chronically low α-KG cannot sustain the demethylation side, which is why ferroptotic susceptibility persists after the initial metabolic insult has resolved.

Second, RNA methylation. *ACSL4* mRNA stability depends on METTL3-mediated m6A methylation, with reader-protein-dependent recognition stabilizing the transcript and sustaining ACSL4 protein and ferroptotic susceptibility across multiple disease contexts (137). Third, histone marks. *ALOX15*—the gene encoding 15-LOX, which partners with PEBP1 to generate hydroperoxy-PE death signals—is itself subject to epigenetic regulation: in disease states, loss of *ALOX15* promoter DNA methylation, driven by EGR1-mediated DNMT1 suppression, raises ALOX15 expression and permits ferroptotic execution (138).

These layers operate on different time scales—DNA methylation over days, m6A on the order of hours, histone marks at intermediate scales—and together they decide a single output: whether a given cell crosses the ferroptotic threshold. This multi-tier architecture makes the prediction stated above falsifiable: the spatial and temporal pattern of cardiovascular ferroptosis is set by the metabolic–epigenetic state of the cell, and ferroptotic death becomes a deferred record of that state.

### 5.2 Role of ferroptosis in cardiovascular diseases

#### 5.2.1 Ferroptosis in atherogenesis and foam cells

**Figure 9:**
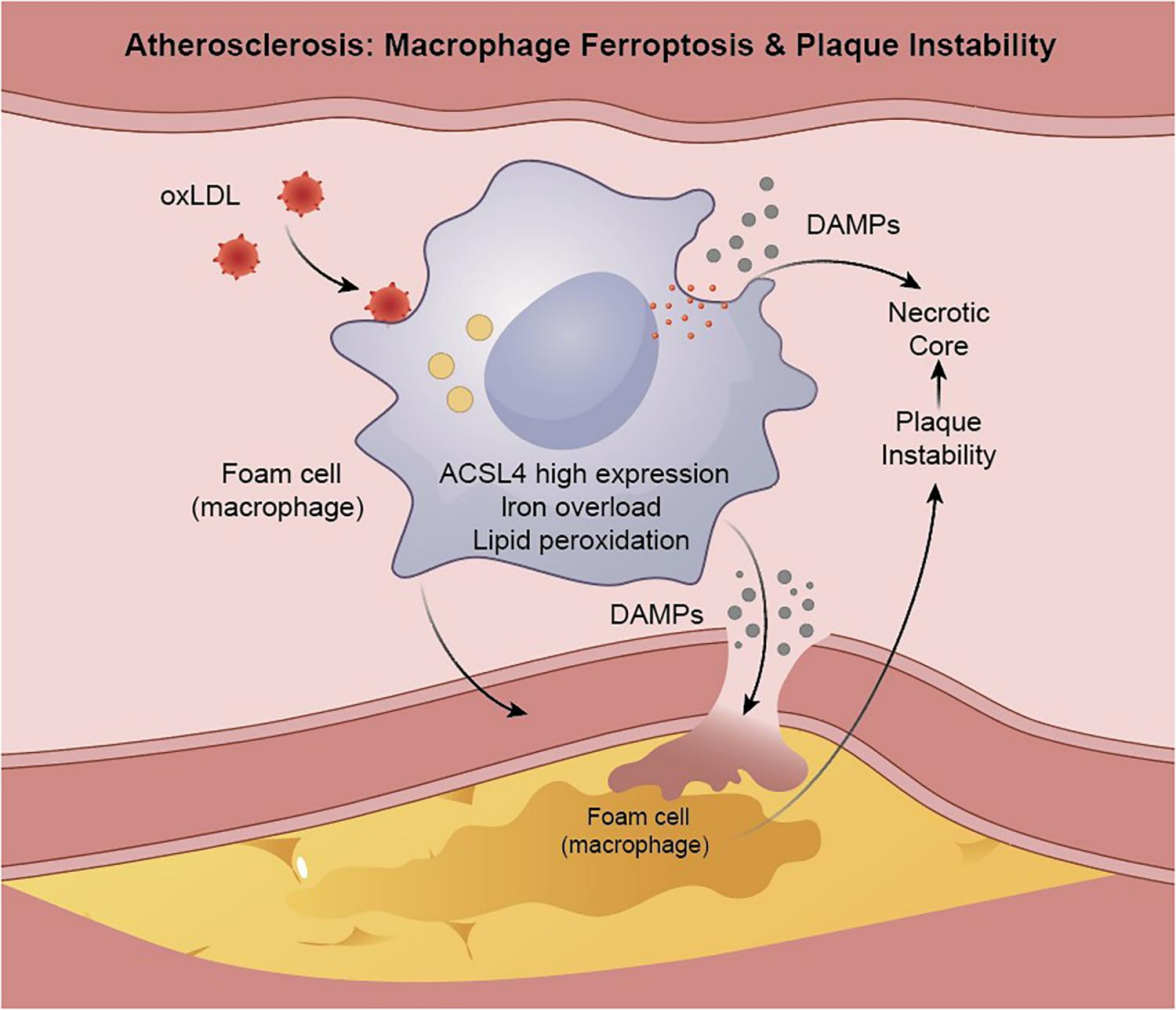
Macrophage ferroptosis drives plaque instability and necrotic core expansion in atherosclerosis. Within atherosclerotic lesions, macrophages internalize oxidized low-density lipoprotein (oxLDL) through scavenger receptors and progressively transform into lipid-laden foam cells. Foam cells exhibit two convergent ferroptosis-promoting features: marked upregulation of acyl-CoA synthetase long-chain family member 4 (ACSL4), which expands the polyunsaturated fatty acid–phospholipid (PUFA-PL) substrate pool, and intracellular iron overload that drives Fenton chemistry-dependent lipid peroxidation. The combined increase in oxidizable substrate and catalytic iron exceeds GPX4 detoxification capacity, triggering ferroptotic execution. Ferroptotic foam cells release damage-associated molecular patterns (DAMPs), free cholesterol, and lipid peroxidation products into the lesion microenvironment, amplifying local sterile inflammation and promoting necrotic core expansion. Because ferroptosis preferentially targets foam cells residing in the lipid-rich necrotic core, the resulting cell loss thins the overlying fibrous cap and shifts plaque morphology toward a rupture-prone phenotype, mechanistically positioning ferroptotic timing at the center of plaque destabilization rather than at its periphery. ACSL4, acyl-CoA synthetase long-chain family member 4; DAMPs, damage-associated molecular patterns; oxLDL, oxidized low-density lipoprotein; PUFA-PL, polyunsaturated fatty acid-containing phospholipid.

Oxidized LDL (oxLDL) is central to atherogenesis (139). It carries lipid peroxidation products, and macrophage uptake of oxLDL triggers both lipid peroxidation and intracellular iron accumulation, ending in ferroptotic cell death. The dying macrophages release pro-inflammatory contents and free cholesterol, which inflame the plaque and promote rupture (140). Foam cells amplify this vulnerability: cholesterol esters stored in lipid droplets sensitize membranes to oxidants, and foam cells upregulate ACSL4, expanding the PUFA-phospholipid pool that lipoxygenases can oxidize (141–143). Foam-cell ferroptosis is not stochastic—it preferentially affects cells in the lipid-rich necrotic core, and the resulting cell loss thins the fibrous cap, placing ferroptotic timing at the center of plaque destabilization rather than at its periphery. Figure 9 shows oxLDL-driven foam-cell ferroptosis, anchored in ACSL4 upregulation and intracellular iron overload, releasing DAMPs and lipid peroxidation products into the necrotic core; the fibrous cap thins, and plaque morphology shifts toward a rupture-prone phenotype.

#### 5.2.2 Ferroptosis as the defining lesion of reperfusion injury

Ischemia–reperfusion (I/R) is the cleanest case in which ferroptosis can be shown as a discrete and clinically targetable lesion. In doxorubicin-exposed mouse cardiomyocytes, ferroptotic features persist after deletion of *Ripk3*, *Mlkl*, or *Fadd*, and ferrostatin-1 outperforms dexrazoxane—the only approved agent for doxorubicin cardiotoxicity—in preventing this damage (120).

The mechanism connects directly to upstream metabolism. The Nrf2–Hmox1 axis accelerates heme degradation under doxorubicin exposure and produces systemic iron overload that concentrates in mitochondria (120). Tadokoro et al. localized the executing lesion to mitochondria themselves: cardiac-specific GPX4 overexpression or mitochondrion-targeted Fe^2+^ chelation prevents lipid peroxidation and cardiomyocyte death in mice (144). The same Nrf2-anchored axis operates in diabetic cardiomyopathy, where AMPK/Nrf2 activation suppresses ferroptosis and rescues cardiac function (145). Across acute and chronic doxorubicin and I/R models, ferrostatin-1, MitoTEMPO, and iron chelators each reduce cardiac dysfunction (146).

**Figure 10:**
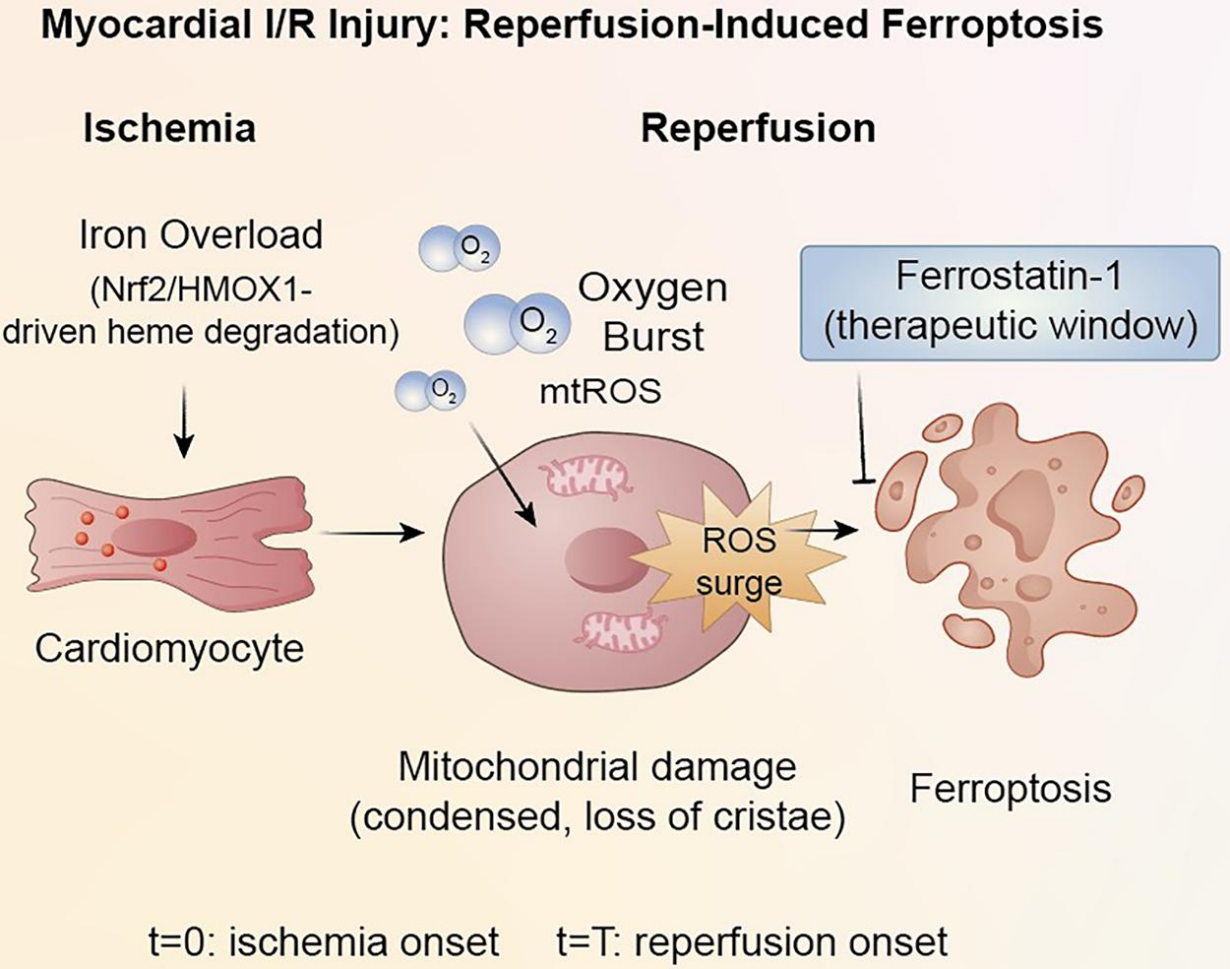
Ferroptosis is a reperfusion-phase, not ischemia-phase, lesion in myocardial ischemia–reperfusion injury, defining a discrete therapeutic window. During the ischemic phase, cardiomyocyte iron loading accumulates—driven by Nrf2–HMOX1-mediated heme degradation and disturbed iron homeostasis—but lipid peroxidation remains restrained by the hypoxic microenvironment. Upon reperfusion, the abrupt restoration of oxygen supply produces an oxygen burst that triggers a mitochondrial reactive oxygen species (ROS) surge: complex I- and complex III-derived superoxide reacts with the pre-loaded labile iron pool through Fenton chemistry, generating hydroxyl radicals that propagate uncontrolled phospholipid peroxidation. The resulting cardiomyocyte ferroptosis is morphologically defined by mitochondrial shrinkage, electron-dense matrix condensation, and loss of cristae integrity. Because lipid peroxidation is initiated specifically by reperfusion-driven oxygen reintroduction rather than by the ischemic insult itself, the ferroptosis inhibitor ferrostatin-1 confers cardioprotection only when administered at or shortly before reperfusion, not during ischemia. This temporal asymmetry defines a precise therapeutic window that reframes the design of cardioprotective trials: pharmacological intervention targeting ferroptotic execution must be temporally aligned with reperfusion onset to be clinically meaningful. I/R, ischemia–reperfusion; ROS, reactive oxygen species.

One clinically decisive observation: ferroptosis is largely a reperfusion-phase event, not an ischemic-phase event (147), as shown in Figure 10. The 15-LOX product 15-HpETE accumulates specifically during prolonged reperfusion and drives PGC1α degradation in cardiomyocytes (148), reinforcing this temporal asymmetry. Pharmacological intervention is therefore meaningful only if delivered at or shortly before reperfusion, not during ischemia—a constraint that defines the therapeutic window and reframes the design of cardioprotective trials.

#### 5.2.3 Ferroptosis in HF: the iron paradox

HF presents an apparent paradox: systemic iron deficiency often coexists with regional myocardial iron accumulation (149). Cardiac-specific ferroportin maintains intracellular iron balance and contractile function, and its loss aggravates iron-driven Fenton chemistry and lipid peroxidation (150). The paradox resolves once ferroptosis is recognized as a cell-autonomous event determined by intracellular labile iron rather than by systemic iron stores. Intracellular labile iron is in turn set by ferroportin transcription, which is governed by the same hypoxia and inflammatory signals discussed above. Iron supplementation in HF must therefore be evaluated against the cellular axis state, not against systemic ferritin alone.

### 5.3 Arachidonic acid metabolism: A single cascade with two epigenetically determined outputs

#### 5.3.1 The COX and LOX pathways

Arachidonic acid (AA) is the dominant membrane PUFA and the precursor for pro- and anti-inflammatory eicosanoids (151). PLA2 releases AA from phospholipids, and downstream enzymes generate diverse bioactive lipids. The cyclooxygenase pathway converts AA to prostaglandins and thromboxanes, with inducible COX-2 producing PGE2 and prostacyclin that support vasodilation and vascular permeability (152). Thromboxane A2 promotes platelet aggregation and vasoconstriction, and aspirin acetylates COX irreversibly, suppressing both inflammatory mediators and thromboxane. The lipoxygenase pathway converts AA to leukotrienes and lipoxins: 5-LOX generates LTA4, the precursor of chemotactic LTB4 and the cysteinyl-leukotrienes that constrict vessels and increase permeability (153).

#### 5.3.2 The resolution program: lipoxins and SPMs

In late inflammation, COX-2 and LOX undergo a metabolic class switch toward pro-resolving mediators (154). Aspirin-modified COX-2 acquires altered substrate specificity and produces 15-HETE, which 5-LOX converts into lipoxins (155); lipoxins inhibit neutrophil recruitment and promote macrophage clearance of apoptotic cells, supporting resolution (156). Specialized pro-resolving mediators (SPMs) extend this program through n-3 PUFA derivatives, with EPA and DHA yielding resolvins, protectins, and maresins (157). SPMs reduce neutrophil infiltration, drive M2 polarization, accelerate efferocytosis, and speed tissue repair. Imbalance between pro-resolving and pro-inflammatory mediators contributes to chronic cardiovascular inflammation and impairs resolution in atherosclerosis and myocardial I/R injury (158); skewed lipoxin-to-leukotriene ratios promote plaque instability (159). After myocardial infarction, rapid SPM synthesis limits inflammation and supports repair (160), and RvD1 reduces infarct size through PI3K/Akt activation in mice (161).

#### 5.3.3 The ferroptotic branch: PEBP1–15-LOX

AA metabolism has a dual character: PEBP1 binds 15-LOX1 and 15-LOX2 and shifts their substrate preference from free PUFAs to membrane phosphatidylethanolamines, generating hydroperoxy-PE death signals (162). When GPX4 cannot detoxify these species, ferroptosis proceeds. The PEBP1–15-LO*X* axis operates in lung epithelium, renal tubular cells, and CNS injury (162). The same enzyme system therefore generates pro-resolving lipoxins under one configuration and ferroptotic death signals under another (163).

#### 5.3.4 The chromatin switch between resolution and death

What configures the same enzyme system toward opposite fates? The answer places AA metabolism inside the metabolic–epigenetic axis, not next to it. The choice is epigenetically gated at the level of substrate-channeling enzyme expression. On the resolution side, p300-mediated H3K27ac at the *PTGS2* promoter sustains COX-2 transcription in macrophages, biasing AA flux toward prostanoid and downstream pro-resolving mediator production. On the death side, *ALOX15* expression is itself epigenetically permitted: loss of DNA methylation at the *ALOX15* promoter raises 15-LOX abundance and, in the presence of PEBP1, drives PE peroxidation toward ferroptotic execution (138). In the heart, *ALOX15* is strongly induced during reperfusion, and its 15-HpETE product triggers cardiomyocyte ferroptosis directly (148). The chromatin state at *PTGS2* vs. *ALOX15* therefore acts as a switch determining whether AA flux runs toward resolution or toward iron-dependent membrane destruction. Figure 8 summarizes this routing: COX-1/2-driven prostanoid signaling; 15-LOX/acetylated COX-2-driven pro-resolving lipoxins and SPMs; 12/15-LOX–PEBP1-driven ferroptotic lipid peroxidation. The resolution-vs.-death decision is set above this routing by the chromatin state at *PTGS2* and *ALOX15*.

### 5.4 Integrated regulation and clinical implications

#### 5.4.1 A time-resolved network

As established in Sections 3 and 4, mitochondrial dysfunction, epigenetic modification, and lipid peroxidation feed back on one another in a time-resolved network rather than as additive layers. Mitochondrial ROS triggers lipid peroxidation, the resulting lipid peroxides damage mitochondrial membranes and the electron transport chain, and the cycle sustains further ROS output. Cytosolic mtDNA activates cGAS–STING and induces type I interferons, while NLRP3-derived IL-1β reshapes neighboring cells' metabolism through autocrine and paracrine signaling (164).

#### 5.4.2 Metabolic memory and the therapeutic implication

**Figure 11:**
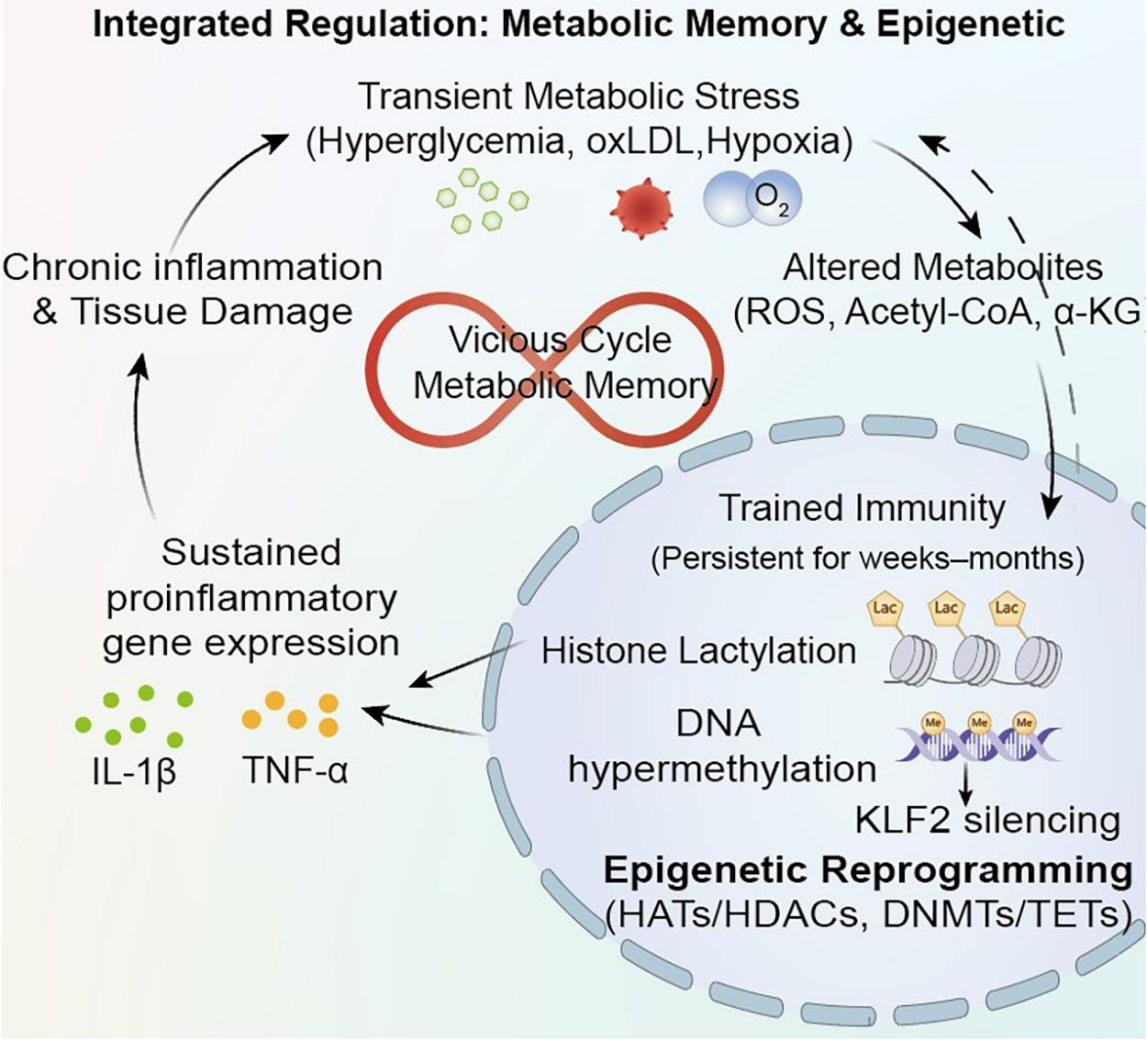
The vicious cycle of metabolic memory: transient metabolic stress imprints durable epigenetic reprogramming that sustains chronic cardiovascular inflammation. Transient cardiovascular metabolic stressors—hyperglycemia, oxidized low-density lipoprotein (oxLDL), and hypoxia—generate altered intracellular metabolite pools, including elevated reactive oxygen species (ROS), nuclear acetyl-CoA, and an imbalanced α-ketoglutarate (α-KG)/succinate ratio. These metabolite shifts drive epigenetic reprogramming through three convergent mechanisms: HAT/HDAC-mediated histone acetylation, p300-catalyzed histone lysine lactylation (Kla, particularly H3K18la), and DNMT/TET-controlled DNA methylation dynamics. The resulting chromatin landscape constitutes the molecular substrate of trained immunity: persistent enrichment of activating histone marks at pro-inflammatory enhancers and DNA hypermethylation at vascular protective gene promoters licenses sustained transcription of pro-inflammatory cytokines (IL-1β, TNF-α). Cytokine-driven chronic inflammation and tissue damage in turn perpetuate metabolic stress, closing a self-reinforcing vicious cycle. Because epigenetic marks remain stable long after the initiating metabolic insult has resolved, this circuit constitutes the molecular basis of “metabolic memory”—the persistence of cardiovascular vulnerability after risk factor normalization—and provides the rationale for therapeutic strategies that simultaneously engage substrate supply, epigenetic enzymes, and downstream inflammatory effectors. α-KG, α-ketoglutarate; DNMTs, DNA methyltransferases; HATs, histone acetyltransferases; HDACs, histone deacetylases; IL-1β, interleukin-1β; Kla, histone lysine lactylation; oxLDL, oxidized low-density lipoprotein; ROS, reactive oxygen species; TETs, ten-eleven translocation dioxygenases; TNF-α, tumor necrosis factor-α.

Epigenetic changes record metabolic history. Transient metabolic shifts produce lasting transcriptional reprogramming through chromatin and DNA-methylation changes (165). This is the mechanism behind “metabolic memory” in diabetes, where prior hyperglycemia keeps patients vulnerable to microvascular disease even after glycemic control is restored. Trained immunity is the analogous phenomenon in macrophages: oxLDL-conditioned monocytes maintain pro-inflammatory programs through stable epigenetic reprogramming (108). Figure 11 shows how transient metabolic stressors (hyperglycemia, oxLDL, hypoxia) imprint durable epigenetic reprogramming through HAT/HDAC-mediated histone acetylation, p300-catalyzed histone lactylation, and DNMT/TET-controlled DNA methylation, producing a persistent loop in which chromatin memory sustains chronic cardiovascular inflammation long after the initiating metabolic insult has resolved.

The therapeutic message is concrete. A single-agent intervention at one node of the axis—a metabolite, a cytokine, or one epigenetic enzyme—is unlikely to be enough, because adjacent layers will quickly restore the inflammatory program from chromatin memory. Combination strategies that act on substrate supply (for example, α-KG or NAD⁺ replenishment), epigenetic enzymes (HDAC or DNMT inhibitors), and the ferroptotic execution machinery (GPX4 stabilization or ACSL4 inhibition) should outperform single-target approaches (166)—a prediction directly testable in the next generation of cardiovascular outcome trials.

## 6 Mechanisms of traditional Chinese medicine intervention in cardiovascular metabolic–inflammatory interactions

If ferroptosis is the deferred readout of an upstream metabolic–epigenetic state rather than an acute injury response, a composite axis biomarker—integrating the succinate/itaconate ratio, H3K18la at inflammatory loci, 5hmC at KLF2, and m^6^A on ACSL4—could prospectively identify cardiovascular patients at imminent ferroptotic risk and define those most likely to benefit from upstream-axis combination therapy.

### 6.1 TCM formula-level modulation of the metabolic–epigenetic axis

Several classical formulas counter the substrate-shift and mitochondrial damage of HF. Shengmai Injection (SMI) activates AMPKα2, up-regulates CPT-1, GLUT-4, and PGC-1α, and preserves mitochondrial respiration and ATP output against angiotensin II–induced cardiomyocyte hypertrophy (167). Shenmai Injection co-activates AMPK and PI3K/Akt/GSK-3β, phosphorylates DRP1-Ser637, and restores L-OPA1/S-OPA1 balance against doxorubicin cardiotoxicity (168). Astragaloside IV (AS-IV), the principal saponin of *Astragalus membranaceus*, activates PPARα, reinstates fatty-acid β-oxidation, raises SERCA2a, and restrains mPTP opening in pressure-overloaded and chronic HF, limiting ventricular remodeling (169, 170).

On the lipid-peroxidation arm, blood-activating formulas suppress ferroptosis. Tanshinone IIA (Tan IIA), from *Salvia miltiorrhiza*, binds VDAC1 to preserve GPX4/GSH and reduce ROS in H/R-injured H9c2 cells (171). Hydroxysafflor Yellow A (HSYA), from *Carthamus tinctorius*, suppresses myocardial I/R ferroptosis via the HIF-1α/SLC7A11/GPX4 axis and protects endothelial cells through miR-429/SLC7A11 signaling in diabetic atherosclerosis (172, 173).

On the inflammation arm, heat-clearing formulas restrain glycolysis–HIF-1α coupling. Berberine (BBR), from *Coptis chinensis*, inhibits mitochondrial complex I and activates AMPK independently of LKB1/CAMKKβ (174), driving GLUT4 translocation and lowering lipid accumulation (175). Baicalin, from *Scutellaria baicalensis*, attenuates I/R injury via PI3K/Akt activation and NF-κB suppression (176), with additional protection through the MAPK pathway (177).

### 6.2 Metabolic regulatory mechanisms of core Chinese herbs

Total ginsenosides from *Panax ginseng* promote cardiomyocyte mitochondrial biogenesis through NAD⁺-dependent SIRT1 activation, with Rg1, Re, Rf, Rb1, Rc, Rh1, Rb2, and Rb3 all acting as SIRT1 activators (178). Ginsenoside Rc binds SIRT1 directly, enhances biogenesis, and up-regulates ETC complexes II–IV (179), while Rg1 triggers SIRT1/PINK1/Parkin-mediated mitophagy (180). *Panax notoginseng* saponins ameliorate diabetic cardiomyopathy by reducing lipid accumulation and restoring mitochondrial structure through modulation of Mfn2, Drp1, and TFAM (181). Tetramethylpyrazine (TMP), the principal alkaloid of *Ligusticum chuanxiong*, protects ischemic myocardium by activating autophagy and suppressing NLRP3 inflammasome assembly, with myosin light chain-2 (Myl2) identified as its direct target (182, 183).

### 6.3 Convergence on the GPX4 axis

Cardiomyocyte ferroptosis during I/R is gated by the SLC7A11/GSH/GPX4 pathway (124). Multiple TCM-derived compounds converge on this axis through distinct mechanisms: salvianolic acid B (Sal B) blocks proteasomal degradation of GPX4 (184). Tan IIA preserves GPX4/GSH while inhibiting VDAC1 (171), HSYA activates HIF-1α/SLC7A11/GPX4 (172, 173), and ginsenoside Rg3 engages the Keap1/Nrf2/GPX4 cascade (185).

### 6.4 Key unresolved scientific questions

**Figure 12:**
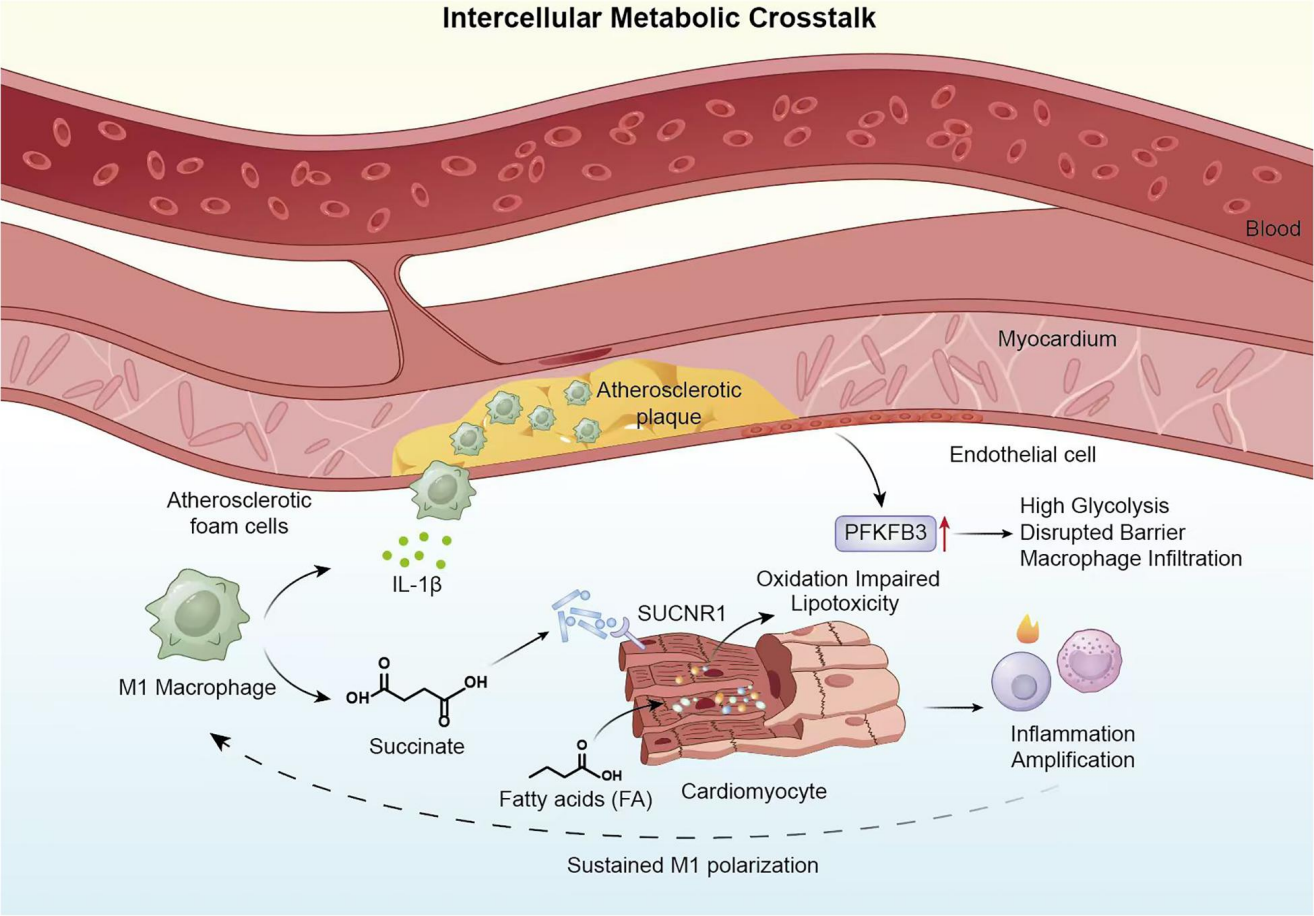
Intercellular metabolic crosstalk amplifies cardiovascular inflammation through paracrine succinate–SUCNR1 signaling, endothelial PFKFB3-driven glycolysis, and cardiomyocyte lipotoxicity. Whereas Figure 1 dissects cell-autonomous metabolic reprogramming in macrophages, endothelial cells, and cardiomyocytes, the present figure integrates these cell-type-specific switches into a system-level network of paracrine metabolic communication. M1-polarized macrophages within atherosclerotic plaques release IL-1β and accumulate succinate, which exits the producing cell and engages succinate receptor 1 (SUCNR1/GPR91) on neighboring cardiomyocytes, exacerbating fatty-acid–driven lipotoxicity in cardiomyocytes with impaired oxidative capacity. Endothelial cells in the inflamed microenvironment upregulate 6-phosphofructo-2-kinase/fructose-2,6-bisphosphatase 3 (PFKFB3), sustaining pathological glycolysis that disrupts barrier integrity and promotes further macrophage infiltration. The three cell-type-specific outputs converge on a self-reinforcing inflammation amplification loop, providing the molecular substrate for the open question of trans-cellular metabolite signaling—whether cardiomyocyte- and macrophage-derived metabolites act in trans to paracrinely reprogram each other's epigenome—and integrating the intracellular metabolic–epigenetic axis defined in Figures 3–11 into a tissue-level inflammatory circuit. EC, endothelial cell; FA, fatty acids; IL-1β, interleukin-1β; PFKFB3, 6-phosphofructo-2-kinase/fructose-2,6-bisphosphatase 3; SUCNR1, succinate receptor 1 (also known as GPR91).

Four questions, each tied to a metabolic–epigenetic node, remain open: (i) whether histone lactylation (e.g., H3K18la at inflammatory promoters) causally drives inflammatory remodeling or is merely a transcriptional readout, which genetic uncoupling of LDHA from p300/EP300 must settle; (ii) whether the metabolic memory of trained immunity—maintained by H3K27ac and H3K4me3 at innate-immune loci—can be erased pharmacologically, and within what therapeutic window; (iii) whether cardiomyocyte- and macrophage-derived metabolites act in trans to reprogram each other's epigenome (e.g., mtDNA leakage activating cGAS–STING in neighboring cells); and (iv) how multi-component formulas converge on a single epigenetic node such as H3K27ac or H3K18la, and whether this convergence is quantifiable by ChIP-seq–based convergence indices. As depicted in Figure 12, paracrine succinate–SUCNR1 signaling, PFKFB3-driven endothelial dysfunction, and lipotoxic cardiomyocyte injury integrate cell-autonomous reprogramming into a tissue-level inflammatory circuit, framing question (iii).

### 6.5 TCM regulation of the metabolic–epigenetic axis

Several compounds map onto the four questions of 6.4, with evidence ranging from cellular and animal models to Phase III trials (summarized in Table 1). Representative single-compound data on pharmacokinetics, effective doses, and safety profiles are summarized in Table 2. BBR restrains the succinate–HIF-1α–IL-1β axis by activating AMPK to suppress HIF-1α and aerobic glycolysis (preclinical; oral bioavailability ∼0.68%) (174, 175, 186). Sal B down-regulates LDHA, lowers nuclear lactyl-CoA, and reduces H3K18la at the LDHA, NLRP3, and IL-1β promoters; LDHA over-expression rescues the inflammatory phenotype, providing genetic evidence for lactylation causality, so far only in non-cardiac tissue (187). Tongxinluo limits cytosolic mtDNA release in a TFAM-dependent manner, dampening cGAS–STING activation; its clinical efficacy is supported by CTS-AMI, the only Phase III RCT in this field (*n* = 3,777 STEMI patients; 30-day MACCE 3.4% vs. 5.2%, RR 0.64, 95% CI 0.47–0.88; benefit sustained at 1 year) (16). Finally, multiple ginsenoside congeners converge on SIRT1, offering a tractable model for quantifying multi-component target convergence by parallel ChIP-seq (178–180, 188). The principal gap is that lactylation causality, erasure of trained-immunity memory, and quantitative target convergence have been demonstrated only in non-cardiac tissues; replication in cardiomyocyte- and cardiac-macrophage-specific models is the most pressing priority. Resolving these gaps will require the kind of AI-driven multi-omics integration that has recently been articulated for natural-product-based precision pharmacology of cardiac remodeling (189), and which we discuss further as a technical bottleneck in section 6.6.

**Table 1:** Traditional Chinese medicine compounds that modulate the metabolic–epigenetic axis in cardiovascular metabolic–inflammatory injury, with axis-node attribution and stratified evidence tier.

Compound (botanical/formula source)	Axis node and key cardiovascular evidence	Evidence tier	Refs
Tongxinluo (multi-herb TCM formula)	Node: mtDNA–cGAS–STING. ↓ cytosolic mtDNA leakage (TFAM-dependent) → dampens cGAS–STING activation in cardiac macrophages. CTS-AMI reduced 30-day and 1-year MACCE in STEMI patients (full trial statistics given in Section 6.2).	Clinical (Phase III RCT)	(16)
Shengmai Injection (*Panax ginseng* + *Ophiopogon japonicus* + *Schisandra chinensis*)	AMPKα2 →↑ CPT-1, GLUT-4, PGC-1α; attenuates Ang II–induced cardiomyocyte hypertrophy and apoptosis.	Cellular + animal	(167)
Shenmai Injection (*Panax ginseng* + *Ophiopogon japonicus*)	Co-activates AMPK and PI3K/Akt/GSK-3β; phosphorylates DRP1-Ser637; restores OPA1; ↓ doxorubicin-induced mtROS and *ΔΨ*m collapse.	Cellular + animal	(168)
Astragaloside IV (*Astragalus membranaceus*)	PPARα → restores FAO; SERCA2a induction; mPTP restraint; ↓ ventricular remodeling in pressure-overload/chronic HF.	Animal	(169, 170)
Tanshinone IIA (*Salvia miltiorrhiza*)	Binds VDAC1 → preserves GPX4/GSH; anti-ferroptotic and anti-apoptotic in H/R-injured H9c2 (docking-confirmed target).	Cellular + animal	(171)
Hydroxysafflor Yellow A (*Carthamus tinctorius*)	HIF-1α/SLC7A11/GPX4 and miR-429/SLC7A11; ↓ myocardial I/R ferroptosis; endothelial protection in T2DM atherosclerosis.	Animal	(172, 173)
Berberine (*Coptis chinensis*)	Inhibits mitochondrial complex I → AMPK activation →↓ HIF-1α/aerobic glycolysis; ↓ atherosclerosis (ApoE−/−); HFpEF mitophagy rescue.	Cellular + animal	(14, 15, 174, 175, 186)
Baicalin (*Scutellaria baicalensis*)	PI3K/Akt activation; NF-κB suppression; ACSL4 inhibition (anti-ferroptosis); ↓ I/R injury; macrophage polarization via JAK/STAT.	Cellular + animal	(176, 177, 191)
Ginsenoside Rc (*Panax ginseng*)	Direct SIRT1 binder → mitochondrial biogenesis (ETC II–IV ↑); ↓ mitochondrial oxidative stress/apoptosis in I/R (EX527-sensitive).	Cellular + animal	(179, 188)
Ginsenoside Rg1 (*Panax ginseng*)	SIRT1/PINK1/Parkin-mediated mitophagy; ↓ cardiac remodeling in HF (EX527/Parkin-knockdown abolish the effect).	Animal	(180)
*Panax notoginseng* saponins (PNS)	Modulates Mfn2/Drp1/TFAM → recouples fission–fusion with biogenesis; ameliorates diabetic cardiomyopathy.	Animal	(181)
Tetramethylpyrazine (*Ligusticum chuanxiong*)	Autophagy activation; Myl2-mediated NLRP3 suppression; protects ischemic myocardium (Myl2 identified as target).	Cellular + animal	(182, 183)
Salvianolic Acid B (*Salvia miltiorrhiza*)	Blocks proteasomal GPX4 degradation; LDHA ↓→ H3K18la ↓ at NLRP3/IL-1β promoters; ↓ myocardial I/R ferroptosis (lactylation causality shown so far in non-cardiac tissue).	Cellular + animal	(184, 187)
Ginsenoside Rg3 (*Panax ginseng*)	Keap1/Nrf2/GPX4 cascade; ↓ myocardial I/R-induced ferroptosis.	Animal	(185)

Evidence tier. Cellular = isolated cells or biochemical/*in silico* assays only. Animal = at least one *in vivo* cardiovascular model (rodent or larger species). Cellular + animal = both layers reported in the cited references. Clinical (Phase III RCT) = a randomized controlled trial powered for hard cardiovascular endpoints (MACE/MACCE/mortality). Tiers are named by experimental system (cellular/animal/clinical) rather than by “*in vitro*/*in vivo*”: *in vitro* denotes cell-free or cell-based systems and *in vivo* denotes intact organisms — animal or human — so neither term is a synonym for “animal experiment.”.

Axis-node attribution. Each compound is mapped to the dominant metabolic–epigenetic node it engages (Sections 3–5): succinate–HIF-1α–IL-1β; itaconate–KEAP1–Nrf2; mitochondrial dynamics (DRP1/MFN/OPA1); mtDNA–cGAS–STING; mtROS–NLRP3 inflammasome; α-KG–TET/JMJD demethylation; lactate–H3K18la lactylation; ACSL4–PUFA membrane remodelling; SLC7A11/GSH/GPX4 ferroptosis execution. Compounds acting at several nodes are noted in the mechanism column.

Caveats. (i) For sodium tanshinone IIA sulfonate (STS) injection and oral berberine, existing Phase II/III cardiovascular trials did not pre-specify these axis nodes as primary readouts; tier ratings therefore reflect mechanism-anchored evidence, not overall clinical evidence. (ii) The H3K18la effect of salvianolic acid B was demonstrated in liver macrophages; direct cardiovascular replication is the priority next step (Section 6.5). (iii) For multi-herb formulas (Tongxinluo, Shengmai, Shenmai), the listed pathway is the dominant validated mechanism; further nodes may emerge with multi-omics, CRISPR and thermal-proteome profiling (Section 6.6).

ACSL4, acyl-CoA synthetase long-chain family member 4; AMPK, AMP-activated protein kinase; cGAS, cyclic GMP–AMP synthase; CPT-1, carnitine palmitoyltransferase 1; DRP1, dynamin-related protein 1; FAO, fatty-acid oxidation; GLUT-4, glucose transporter 4; GPX4, glutathione peroxidase 4; GSH, glutathione; HF, heart failure; HFpEF, heart failure with preserved ejection fraction; HIF-1α, hypoxia-inducible factor 1α; H3K18la, histone H3 lysine-18 lactylation; IL-1β, interleukin-1β; I/R, ischemia–reperfusion; KEAP1, Kelch-like ECH-associated protein 1; LDHA, lactate dehydrogenase A; MACCE, major adverse cardiac and cerebrovascular events; MACE, major adverse cardiovascular events; MFN, mitofusin; mPTP, mitochondrial permeability transition pore; mtDNA, mitochondrial DNA; mtROS, mitochondrial reactive oxygen species; NLRP3, NOD-like receptor family pyrin domain-containing 3; Nrf2, nuclear factor erythroid 2-related factor 2; OPA1, optic atrophy 1; PGC-1α, PPAR-γ coactivator 1α; PPARα, peroxisome proliferator-activated receptor α; RCT, randomized controlled trial; SERCA2a, sarco/endoplasmic reticulum Ca^2+^-ATPase 2a; SIRT1, sirtuin 1; SLC7A11, solute carrier family 7 member 11; STEMI, ST-segment elevation myocardial infarction; STS, sodium tanshinone IIA sulfonate; TFAM, mitochondrial transcription factor A; T2DM, type 2 diabetes mellitus; VDAC1, voltage-dependent anion channel 1.

**Table 2:** Pharmacokinetics, effective doses, and safety profiles of representative TCM-derived single compounds.

Compound	Source/class	Effective preclinical dose	Clinical dose	Oral bioavailability	Safety/toxicity profile	References
Berberine (BBR)	Isoquinoline alkaloid; *Coptis chinensis* (Huang Lian); heat-clearing class	50–200 mg/kg/day, oral gavage (mice/rats): in the TAC-induced heart-failure model, 50 mg/kg/day was sufficient to upregulate PINK1/Parkin-mediated mitophagy and preserve cardiac function; in the HFpEF model, 100 and 200 mg/kg/day improved mitochondrial homeostasis and suppressed apoptosis through AMPK/PGC-1α signaling	0.9–1.5 g/day, given orally in three divided doses (T2DM/dyslipidemia RCTs); 1.5 g/day represents the common clinical upper limit	Absolute oral bioavailability ≈ 0.68% in rats (100 mg/kg p.o.); limited by P-gp efflux and CYP-mediated first-pass metabolism	Generally well tolerated. The most common adverse events are mild, transient gastrointestinal symptoms (diarrhea, constipation, abdominal distension, abdominal pain). In a 13-week T2DM clinical trial, ALT, γ-GT, and creatinine showed no significant changes and no serious adverse events were reported	(192–195)
Tanshinone IIA (Tan IIA)/Sodium Tanshinone IIA Sulfonate (STS, also DS-201)	Diterpenoid quinone; *Salvia miltiorrhiza* (Dan Shen); blood-activating class	Tan IIA: 5–70 mg/kg administered i.v. or i.p. in rat/mouse models of myocardial ischemia–reperfusion, myocardial infarction, and pressure-overload heart failure (CAMARADES systematic review of 28 studies)	STS (water-soluble derivative): 40–80 mg/day by intravenous infusion (NMPA-approved injectable in China; standard cardiovascular regimen)	Free Tan IIA shows poor oral bioavailability, with an absolute bioavailability <3.5%, attributable to high lipophilicity, low aqueous solubility, P-gp efflux, and hepatic first-pass metabolism; STS is administered intravenously to bypass these limitations	In a meta-analysis of STS injection for lipid modulation in coronary heart disease, the safety profile was favorable, with no significant increase in adverse events vs. controls. A recent comprehensive review concludes that Tan IIA exerts multi-target cardioprotection across diverse cardiovascular models, with no consistent dose-dependent hepatotoxicity reported	(196–200)
Ginsenoside Rc	Protopanaxadiol (PPD)-type tetracyclic triterpenoid saponin; *Panax ginseng* (Ren Shen); tonifying class	10–40 mg/kg/day, oral gavage (mice): in the isoproterenol-induced myocardial ischemia model, 10, 20, and 40 mg/kg conferred antioxidative and anti-inflammatory protection through Nrf2/HO-1 signaling; in ApoE⁻^/^⁻ mice on a high-fat diet (anti-atherosclerosis model), 40 mg/kg/day modulated the gut microbiota and the fecal metabolite profile	No approved clinical formulation of Rc as a single compound is currently available; human dosing has not been established for Rc *per se*, since systemic exposure is achieved primarily through ingestion of ginseng/red ginseng extracts containing Rc	Absolute oral bioavailability ≈ 0.17% in rats (20 mg/kg p.o. vs. 2 mg/kg i.v.); as a polyglycosylated PPD-type saponin, intestinal absorption is poor (<5%), and *in vivo* activity is largely attributable to the deglycosylated gut-microbiota metabolite Compound K	In published cardioprotection and anti-atherosclerosis studies, doses of 10–40 mg/kg/day produced no overt organ toxicity. Because Rc is typically administered as part of a ginseng matrix, dedicated human safety data are limited. Compound K, derived from Rb1, Rb2, and Rc by intestinal microbial transformation, has been generally well tolerated when delivered as a red ginseng extract in healthy volunteers, but human pharmacokinetic and safety data for Rc as a single compound remain insufficient	(201–205)

AE, adverse event; ALT, alanine aminotransferase; AUC, area under the curve; BBR, berberine; CHD, coronary heart disease; CYP, cytochrome P450; DCM, diabetic cardiomyopathy; HF, heart failure; HFpEF, heart failure with preserved ejection fraction; i.p., intraperitoneal; i.v., intravenous; I/R, ischemia–reperfusion; MI, myocardial infarction; NMPA, National Medical Products Administration (China); p.o., per os (oral); P-gp, P-glycoprotein; PCI, percutaneous coronary intervention; PPD, protopanaxadiol; RCT, randomized controlled trial; STS, sodium tanshinone IIA sulfonate; TAC, transverse aortic constriction; T2DM, type 2 diabetes mellitus; Tan IIA, tanshinone IIA; γ-GT, γ-glutamyl transferase.

### 6.6 Limitations and technical challenges

Three technical bottlenecks restrain progress.

First, **dynamic monitoring of metabolites in living tissue.** The immunometabolites that anchor the axis defined in Sections 3 and 4 — the succinate–HIF-1α–IL-1β circuit (3), the itaconate–KEAP1–Nrf2 and itaconate–NLRP3 circuits (5, 93), and the α-KG/succinate-ratio switch governing macrophage polarization (29) — operate across cytosol, matrix, and nucleus on the seconds timescale that defines the ischemia-to-reperfusion transition. Conventional mass-spectrometry-based metabolomics requires tissue homogenization and so collapses these spatial and temporal gradients. Genetically encoded biosensors with subcellular targeting would resolve this, yet validated sensors for succinate, itaconate, and α-KG remain undeveloped. This gap is the principal methodological obstacle to settling open question (i) of Section 6.4 — the causal status of histone H3K18la lactylation (13, 117)— in cardiac-specific models, because settling that question requires real-time tracking of nuclear lactate flux relative to lactyl-CoA supply, exactly the measurement that current technology cannot yet make.

Second, **single-cell metabolomics is immature.** Although single-cell RNA-seq has already resolved aortic macrophages into resident-like, IL-1β-high, and TREM2⁺ lipid-handling subsets in atherosclerosis (30), mass-spectrometry sensitivity for metabolites lags single-cell transcriptomics by 1–2 orders of magnitude, and metabolites cannot be amplified. Resolving the intratissue metabolic heterogeneity highlighted in Sections 2 and 4 — and in particular the paracrine succinate–SUCNR1 (92), PFKFB3-driven endothelial (33), and lipotoxic-cardiomyocyte triad shown in Figure 12 — will require coordinated advances in single-cell mass-spectrometry sensitivity together with metabolic flux inference applied to cardiac single-cell datasets.

Third, **multi-target attribution of traditional Chinese medicine formulas.** Conventional pharmacology cannot pinpoint which compound–target pair drives the clinical efficacy of multi-component formulas, exemplified by Tongxinluo in CTS-AMI — the only Phase III randomized trial discussed in Section 6.5 (16) — and by Shengmai (167) and Shenmai (168) injections [Table 1, footnote (iii)]. A tractable framework would integrate AI-driven network pharmacology to nominate candidate compound–target pairs, genome-wide CRISPR screens to test functional necessity at the axis nodes defined in Sections 3–5, and thermal proteome profiling to confirm physical target engagement. Quantitative target-convergence indices — obtained, for example, by parallel ChIP-seq of whole formulas vs. their dominant single compounds [berberine (14, 15) within *Coptis*-containing decoctions; salvianolic acid B (184, 187) within *Salvia*-based preparations, as developed in Section 6.5 and Table 1], or by extending the ginsenoside-on-SIRT1 convergence model already developed at Section 6.5 (178–180, 188) — would replace qualitative “multi-target” descriptors with falsifiable convergence measurements, This convergence framework is conceptually aligned with the recently proposed paradigm of integrating AI-driven analytics with multi-omics data to rationalise the multi-target pharmacology of natural products in cardiovascular disease (189), in which single-cell spatial transcriptomics and machine learning are used to map ginsenoside- and baicalin-type compounds onto pathway-level targets such as AMPK activation and NF-*κ*B suppression, directly addressing open question (iv) of Section 6.4.

## 7 Conclusions and future perspectives

This review consolidates evidence that cardiovascular inflammation is governed by a unified metabolic–epigenetic axis in which TCA-cycle intermediates both fuel bioenergetics and directly regulate chromatin-modifying enzymes (1, 190). Succinate accumulation stabilizes HIF-1α and drives IL-1β transcription, whereas IRG1/ACOD1-derived itaconate opposes this circuit by inhibiting succinate dehydrogenase (4) and disrupting NLRP3 assembly (93); glycolysis-derived nuclear acetyl-CoA supplies the H3K27ac substrate for trained immunity (104), through which a transient Western-diet stimulus imprints durable inflammatory phenotypes that persist after the stimulus is withdrawn (109). The axis terminates in SLC7A11/GSH/GPX4-gated ferroptosis, which executes this upstream dysregulation in atherogenesis, ischemia–reperfusion injury, and HF (120). Mitochondrial dynamics, mtDNA-driven cGAS–STING activation, and NLRP3-mediated cytokine release are therefore not parallel injury pathways but convergent outputs of a single regulatory node defined by metabolite availability and chromatin state.

Three questions will determine whether this framework yields actionable therapy: the causal status of histone lactylation, which requires genetic uncoupling of LDHA from p300/EP300 in cardiac-specific models; cell-type-selective delivery, since succinate buffering, α-KG repletion, and ferroptosis inhibition produce opposite consequences across cardiovascular cell types and demand platforms such as nanoparticles, aptamers, or exosomes; and the mechanistic dissection of multi-component TCM formulas, where parallel ChIP-seq of whole extracts vs. single compounds could replace qualitative “multi-target” claims with falsifiable convergence measurements. Gut-microbiota–derived metabolites, single-cell metabolomic resolution of intratissue heterogeneity, and the temporal hierarchy among DNA methylation, m⁶A, and histone modifications are adjacent priorities that will refine, but not displace, these three central questions.

Clinical translation has passed proof-of-principle, with three landmark trials validating distinct nodes of the same axis: canakinumab (anti–IL-1β) reduced MACE independent of LDL lowering in CANTOS (8); low-dose colchicine, acting upstream at tubulin–NLRP3 assembly, reduced MACE in COLCOT and LoDoCo2 (9, 10); and the multi-component formula Tongxinluo lowered 30-day and 1-year MACCE in STEMI patients in CTS-AMI, plausibly through TFAM-dependent suppression of mtDNA-driven cGAS–STING signaling (16). The decisive next step is biomarker-guided stratification—classifying prospective cohorts by composite axis signatures such as the circulating succinate-to-itaconate ratio, peripheral-monocyte H3K18la, and vascular 5hmC at KLF2—to identify patients most likely to benefit from upstream-axis combination therapy. This precision-immunometabolic strategy, integrating mechanism-defined Western pharmacology with mechanism-anchored TCM compounds, defines the agenda for the next generation of cardiovascular outcome trials.
